# Essential components and pathways for developing Indigenous community‐based monitoring: Examples from the Canadian oil sands region

**DOI:** 10.1002/ieam.4485

**Published:** 2021-08-03

**Authors:** Danielle Beausoleil, Kelly Munkittrick, Monique G. Dubé, Faye Wyatt

**Affiliations:** ^1^ Alberta Environment and Parks, Alberta, Canada; ^2^ University of Calgary, Calgary, Alberta, Canada; ^3^ Cumulative Effects Environmental Inc., Alberta, Canada; ^4^ UK Met Office, UK

**Keywords:** Indigenous community‐based monitoring, Indigenous knowledge, Integration, Oil sands, Section 35 Rights

## Abstract

Historically, environmental research and monitoring in the Alberta oil sands region (OSR) located in northeastern Alberta, Canada, have largely neglected, meaningful Indigenous participation. Through years of experience on the land, Indigenous knowledge (IK) holders recognize change on the landscape, drawing on inextricable links between environmental health and practicing traditional rights. The cumulative impacts of crude oil production are of great concern to Indigenous communities, and monitoring initiatives in the OSR provide unique opportunities to develop Indigenous community‐based monitoring (ICBM). A review of ICBM literature on the OSR from 2009 to 2020 was completed. Based on this review, we identify best practices in ICBM and propose governance structures and a framework to support meaningful integration of ICBM into regulatory environmental monitoring. Because it involves multimedia monitoring and produces data and insights that integrate many aspects of the environment, ICBM is important for natural science research. ICBM can enhance the relevance of environmental monitoring by examining relationships between physical and chemical stressors and culturally relevant indicators, so improving predictions of long‐term changes in the environment. Unfortunately, many Indigenous communities distrust researchers owing to previous experiences of exploitive use of IK. In the present paper, we recommend important practices for the integration of IK into regional environmental monitoring programs. ICBM is important to communities because it includes conditions to which communities can exercise traditional rights, and highlight how industrial activities affect this ability. Equally important, ICBM can generate a resurgence of Indigenous languages and subsequently traditional practices; it can also revive the connection with traditional lands and improve food security. *Integr Environ Assess Manag* 2022;18:407‐427. © 2021 The Authors. *Integrated Environmental Assessment and Management* published by Wiley Periodicals LLC on behalf of Society of Environmental Toxicology & Chemistry (SETAC).

## KEY POINTS

- The paper provides a unique way of organizing the integration of Indigenous knowledge (IK) and Western science (WS) using a conceptual model, which can allow regional monitoring programs to constantly evaluate research objectives and identify gaps.
- It is not the intention of this paper to adapt IK or WS to any model or monitoring program, but to discuss the integrative ability of both to better understand pressure/stressor‐pathways and response relationships of environmental impacts in the oil sands region.
- Contextualizing each program into the conceptual model is useful for large monitoring programs, such as the Oil Sands Monitoring (OSM) program, to integrate large amounts of data assessing different aspects of the environment over time and space.
- Indigenous community‐based monitoring approaches, such as those led by the Mikisew Cree and Athabasca Chipewyan First Nations, support regional monitoring where data are easily captured in regional‐scale models.

## INTRODUCTION

The oil sands region (OSR) located in Northern Alberta, Canada, encompasses approximately 11 million acres (4.3 million hectares), an area roughly the size of the US state of Florida. The ground beneath this region is a mixture of sand, clay, and a heavy crude oil or tarry substance known as bitumen, collectively referred to as tar sands or oil sands. Considerable amounts of water and land are required for oil sands extraction. The consequences to the Athabasca River watershed have been the focus of several decades of environmental monitoring by the provincial and federal Canadian governments, as well as industry. Industrial development creates concerns among Indigenous peoples regarding the safety of consuming traditional resources, such as wild game, and the quality of water and air (Bill et al., 1996). Marginalized communities, particularly Indigenous communities, often experience disproportionate burdens of environmental pollution (Fernandez‐Llamarzares et al., 2020). The ecological disruption and presence of environmental contaminants have prevented many Indigenous peoples from practicing traditional lifestyles (Dennis et al., 2015; Hoover et al., 2012). Research suggests that these consequences are a result of colonization and historical exclusion of Indigenous concerns in the development of polluting infrastructures, built without the free, prior, and informed consent of affected communities (Fernandez‐Llamarzares et al., 2020).

Monitoring in the OSR provides unique opportunities to develop Indigenous community‐based monitoring (ICBM). ICBM utilizes both Indigenous knowledge (IK) and Western science (WS), combining the strengths of both to tackle challenging environmental issues. Lessons can be learned from experiences involving Indigenous people in the planning, development, and integration of ICBM into regional monitoring programs. In the OSR and elsewhere in Canada, IK is maintained, transmitted, and developed by Indigenous peoples through lived experience on the land, exercising Section 35 Rights of the Constitution Act of Canada (such as hunting, fishing, trapping, gathering, harvesting, and ceremonial, cultural, recreational, and domestic uses) over many generations (Candler et al., 2010; Government of Canada, 1968–1982). Inextricable linkages between environmental health and the practice of Section 35 Rights allow knowledge holders to recognize changes in the environment. ICBM is driven by Section 35 Rights. To adequately address concerns raised by Indigenous communities, they must be involved in all aspects of monitoring design and analysis, and reporting is required to ensure that the monitoring program meets all needs (Dubé et al., 2021).

Initiated by the experiences of residential school survivors, the Truth and Reconciliation Commission of Canada (TRCC) released 94 Calls to Action to help facilitate reconciliation between Indigenous and non‐Indigenous peoples in Canada (TRCC, 2015). Although this has been an important step towards reconciliation, these Calls to Action largely target governments and institutions and do not provide the tools necessary for individuals, such as scientists, to engage meaningfully with Indigenous peoples. Recently, involving Indigenous people in environmental monitoring programs has started to become more common. Understanding how information is generated in ICBM and integrated into monitoring programs is equally meaningful. Avoiding misinterpretation of IK requires approaches that support the context, reason, and meaning behind the information, including the relationship to the land and connection to place and language. Equitably and ethically integrating and maintaining the integrity of different knowledge systems can be challenging. Throughout this paper, we highlight 10 Calls to Action recently described for researchers in natural science when engaging with Indigenous peoples in environmental monitoring (Table 1; Wong et al., 2020). These Calls to Action represent a greater responsibility in monitoring design that can improve the development and integration of ICBM in regional monitoring programs and improve WS programs that involve Indigenous peoples.

**Table 1:** Ten calls to action directed towards natural science researchers can help to improve Indigenous community‐based monitoring programs and Western science‐based monitoring programs that involve Indigenous people

	Ten (10) Calls To Action–Directed Towards Natural Science Researchers
Call to Action 1	We call on natural scientists to understand the socio‐political landscape around their research sites.
Call to Action 2	We call on natural scientists to recognize that generating knowledge about the land is a goal shared with Indigenous peoples and to seek meaningful relationships and possible collaboration for better outcomes for all involved.
Call to Action 3	We call on natural scientists to enable knowledge sharing and knowledge co‐production.
Call to Action 4	We call on natural scientists studying animals to seek out advice from Elders for respectful ways of handling animals.
Call to Action 5	We call upon natural scientists to provide meaningful opportunities for Indigenous community members, particularly youth, to experience and participate in science.
Call to Action 6	To decolonize the landscape, we call on natural scientists to incorporate Indigenous place names as permitted.
Call to Action 7	We call upon natural scientists and their students to take a course on Indigenous history and rights.
Call to Action 8	We call on funding bodies to change approaches to funding.
Call to Action 9	We call on editors of all scientific journals to recognize that publication of research on Indigenous Knowledge and cultural resources require review and permission from the respective Indigenous communities.
Call to Action 10	Finally, we call on all natural scientists and postsecondary research institutions to develop a new vision for conducting natural science: fundamentally mainstreaming reconciliation in all aspects of the scientific endeavor, from formulation to completion.

In 2017, the governments of Alberta and Canada included a commitment to enhance the role of ICBM in the oil sands regional monitoring program with improved capacity building and a memorandum of understanding (Environment and Climate Change Canada, 2018). Working collaboratively with the governments of Alberta and Canada, 18 Indigenous communities (and regions) in the OSR subsequently participated in the development of an Operational Framework Agreement (OFA). The OFA, developed to guide the governance of the joint Alberta–Canada Oil Sands Monitoring (OSM) program, strives to improve Indigenous participation, transparency, and inclusion of IK (Dubé et al., 2018). The OSM program involves multimedia monitoring and producing data on many aspects of the environment. Indigenous people view the environment as a holistic ecosystem. As such, environmental considerations of ICBM can enhance the relevance of environmental monitoring strategies across multiple media and produce data on many aspects of the environment. This is important for natural scientists (scientists studying the environment) when examining stressor‐pathway and response relationships that involve multiple environmental media and knowledge systems. ICBM can fill relational gaps between both physical and chemical stressors (landscape disturbance and contaminants) and culturally relevant indicators observed in the environment by Indigenous peoples. Important to communities, ICBM can assess conditions to which they can exercise Section 35 Rights and describe how industrial activities affect this ability. Research done in this way becomes a reciprocal process and can improve relationships between scientists and communities because everyone benefits from the experience (Baker, 2016; McGregor, 2018).

As part of a media‐themed series of the environmental monitoring literature in the OSR, a review of ICBM literature in the OSR from 2009 through 2020 was completed. Based on this review, we first discuss IK and language. Deeply rooted in stewardship and environmental governance, language is very important to consider in ICBM. In this context, ICBM is not simply monitoring what remains but also helping to restore what was lost. We then discuss governance structures and framework to support meaningful integration of IK and ICBM into regulatory environmental monitoring. Using a WS perspective, we use a conceptual model to organize ICBM literature along a pressure/stressor‐pathway‐response relationship that involves several environmental media. As a visual guide to highlight monitoring gaps and improve data integration, conceptual models can help form hypotheses about cause–effect relationships that are observed in the environment from oil sands related activities. Finally, we identify types of ICBM and review ICBM and WS programs that involve Indigenous communities in the OSR region. We focus on best practices to improve future ICBM and WS programs involving Indigenous people in regional monitoring programs.

## INDIGENOUS KNOWLEDGE, LANGUAGE, AND ICBM

Indigenous knowledge refers to a collective understanding of a particular environment, passed down from generation to generation by the Indigenous peoples living in a particular landscape for hundreds to thousands of years (Porten et al., 2016). IK includes information on the state of the ecosystem and how environmental dynamics change over time (Johnson et al., 2016). Passed along to the next generation, IK is validated by testing in practice what is found to be relevant and consistent over time (Inuit Circumpolar Council, 2013). IK can provide indicators that are useful to communities, researchers, government agencies, nongovernmental organizations, and other interested parties when assessing change in particular ecosystems (Johnson et al., 2016).

Health research in parts of Canada and Australia has identified links between Indigenous languages and the health, wellness, and personal self‐esteem of Indigenous peoples. This is potentially a mark of cultural persistence, possibly connected by the transmission of IK and values (Corntassel & Hardbarger, 2019; Dockery, 2012; Ferguson & Weaselboy, 2020; Hallett et al., 2007; McIvor, 2013). Indigenous languages are the foundation to culture and identity that bonds communities to thousands of years of history, deeply rooted in Indigenous culture, law, and environmental governance (Ebel, 2014; Enns et al., 2018). From maintaining stewardship of land for millennia, Indigenous peoples are driven by self‐sustaining ambitions to utilize resources they in turn have a responsibility to sustain (Low & Shaw, 2011; Tran et al., 2020). Environmental stewardship reflects the history, culture, and law of Indigenous communities and is evident in the ways they design and implement their activities (Reed et al., 2020). Recently, the value of IK and language to environmental monitoring practices has become more recognized by Western scientists.

The use of place‐names is important to Indigenous language and culture. Indigenous place‐names can contain references to place‐based plants and animals and can provide distribution, migration, and behavior patterns of species and their local uses (Wong et al., 2020). Indigenous place‐names can even mark locations of significance, include geographical features, or warn you of potential danger. Place‐names can tell stories and provide clues about the landscape, describe how people lived and associated with that landscape, and help to reaffirm the Indigenous language that evolved there (Henshaw, 2006; Sousa et al., 2010; Wong et al., 2020). The process of passing knowledge through the use of place‐names inextricably connects Indigenous people with their environment (Wilder et al., 2016). The concept of passing knowledge through language becomes a process that depends on opportunities to engage on that land over many generations (Ferguson & Weaselboy, 2020). If changes occur on landscapes, place‐names and language stories associated with those landscapes may no longer reflect the same meaning. This can increase the difficulty of preserving knowledge in those places. Call to Action 6 (Table 1) calls for the decolonization of the landscape, supported by the recognition and use of Indigenous place‐names (Wong et al., 2020). Language connects the use of place‐names and the resurgence of Indigenous cultures. Indigenous peoples of Canada are working to restore place‐names in part to revitalize their languages after colonial policies and legislation have attempted to eradicate them (Gray & Rűck, 2019). Recently, we have seen more efforts in parts of Canada to undertake ICBM place‐name projects to strengthen the presence of Indigenous culture(s) and help restore language and knowledge in the area (Gray & Rűck, 2019; Tracking Change, 2016). To improve best practices in ICBM, efforts should include the use of place‐names or place‐naming activities.

## INDIGENOUS COMMUNITIES, OPERATIONAL FRAMEWORK, AND KEY PRACTICES FOR ICBM IN THE OSR

**Figure 1:**
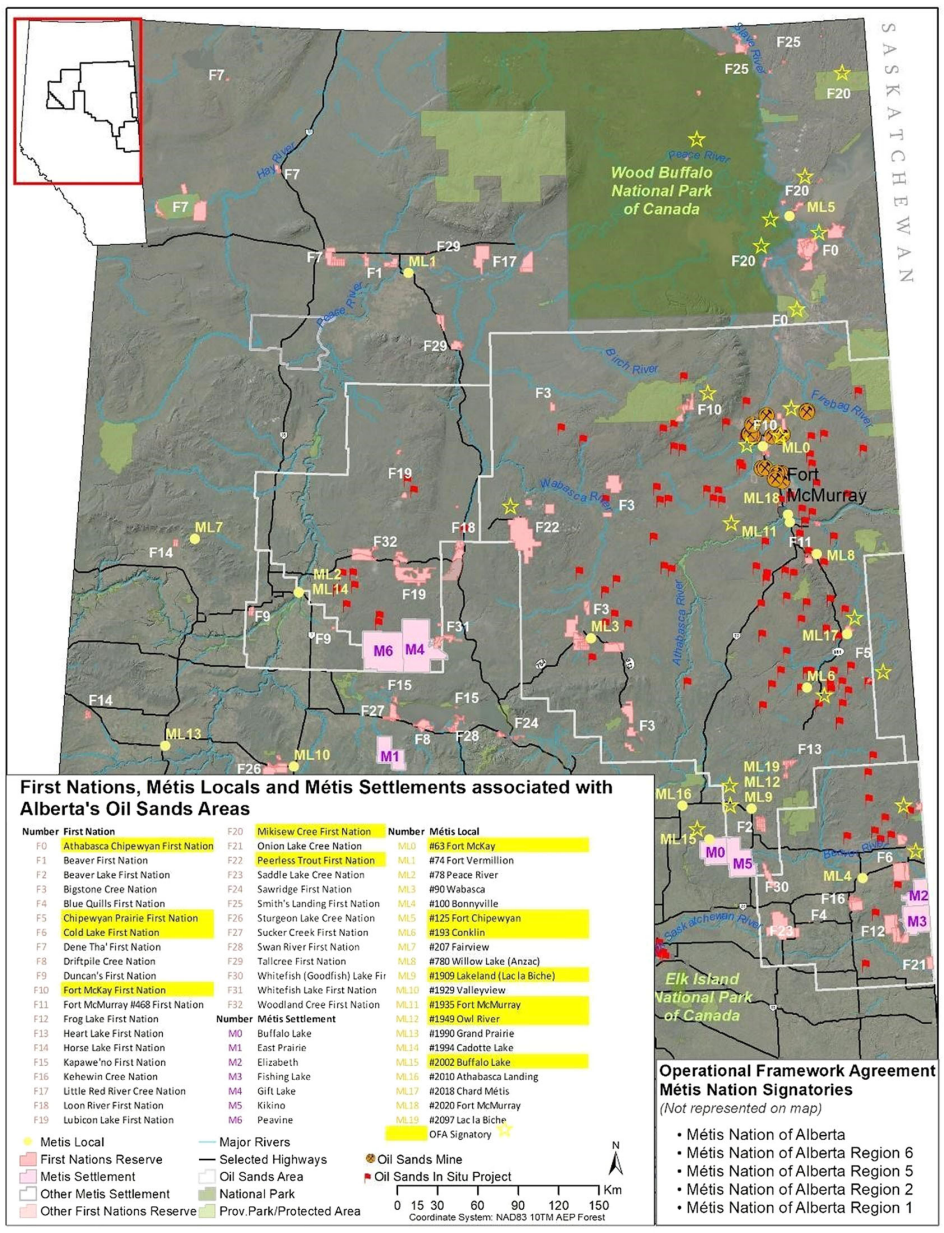
Map of First Nations, Métis locals, and Métis settlements associated with Alberta's oil sands areas. The communities highlighted in yellow formed part of the OSM program governance structure and participated in the development of the Operational Framework Agreement to guide the program

Approximately 23 000 Indigenous people from 18 First Nations and six Métis settlements live in the oil sands regions of Alberta (Government of Canada, 2016). Figure 1 includes a map of all 41 First Nations and Métis locals and settlements located within and surrounding the Alberta oil sands areas relative to oil sands operations. The OFA to guide the governance of the OSM program in the OSR was developed with the active participation of the 18 First Nations and Métis, which are highlighted in Figure 1 (Dubé et al., 2018). Although the OFA began implementation in 2019, more time is required to allow the full design to come to fruition, including authentic and truly inclusive Indigenous participation. It remains to be seen how or if political influences will affect the program (Dubé et al., 2021).

The current framework of the OSM program involved the formation of an ICBM Advisory Committee (ICBMAC) to help facilitate Indigenous participation in OSM programs and advise a Science and Indigenous Knowledge Integration Committee (SIKIC). The ICBMAC can play a key role in fostering ethical integration of IK and community‐developed, culturally relevant indicators in OSM programs, including sociocultural and sociopolitical components that typically fall outside WS monitoring programs, such as accessibility of Section 35 Rights. The SIKIC, in turn, evaluates recommendations for funding OSM programs, including ICBM and capacity‐building programs, and provides annual recommendations to the OSM program cochairs on funding allocation (Dubé et al., 2018). As such, monitoring programs need to support OSM objectives and priorities as outlined in the OFA. In addition to any funding provided annually through OSM work plans, the government of Canada provides additional support for capacity‐building initiatives within Indigenous communities (Government of Alberta, 2018).

The term capacity, defined in the context of Indigenous program delivery, is the availability of funding and capability of Indigenous communities to manage tasks associated with participating in government programs and services (Nicolas Applied Management Inc., 1996). Further, to ensure consistency and the ability to integrate data across a regional monitoring program, capacity can also refer to the ability to follow specific standardized quality assurance and quality control (QA/QC) and standard operating procedures (SOPs). However, capacity in Indigenous program delivery can and should also be defined as experience with ICBM methodologies, local knowledge, scientific skills, safety skills, technical skills, communication skills, holistic oversight, and project management (Johnson et al., 2016). The level of capacity for Indigenous people to participate in WS activities (Table 1, Call to Action 5) is just as important as the capacity of researchers to participate using Indigenous methodologies (Table 1, Call to Action 4and Call to Action 7). Best practices of ICBM include building capacity for communities and natural science researchers.

Capacity building is a long‐term endeavor that requires stable funding, training, and outreach to youth, Elders, and interested community members to improve skills and competencies in science and technology, and to strengthen cultural experiences to preserve IK over generations. It also requires a shift in WS‐driven programs to offer space for meaningful collaboration with Indigenous communities. Although WS‐driven programs involving Indigenous people are not true ICBM (types are discussed later), they can still offer a starting point to increasing capacity in communities who may want to develop their own ICBM programs that use both IK and WS. Best practices in ICBM will encourage meaningful opportunities for community members, especially youth, to participate in science, as this can facilitate capacity for ICBM over generations (Wong et al., 2020). This is especially important as it relates to resource access and human health implications where communities are concerned about consuming traditional food and medicines (i.e., Section 35 Rights) in the OSR (Joly & Westman, 2019). In the context of the OFA, objectives to increase capacity have advanced the OSM program in terms of improving Indigenous engagement and participation, including the perspectives and concerns of Indigenous communities in all aspects of the program, from onset to completion (Table 1, Call to Action 10; Wong et al., 2020).

The level of community capacity can vary from community to community in the OSR, which can influence development of ICBM. Some communities among the most experienced in ICBM include the Mikisew Cree First Nation (MCFN) and the Athabasca Chipewyan First Nation (ACFN). The MCFN and ACFN live downstream of oil sands industries, along the Athabasca River and into the Peace–Athabasca Delta (PAD). As a complex and ecologically diverse freshwater delta, PAD is very important to Indigenous communities. The MCFN, discussed next, are well known for their involvement in environmental monitoring strategies in the OSR and have greatly influenced ICBM and the integration of IK in regional monitoring practices (Parks Canada, 2021; United Nations Educational, Scientific and Cultural Organization [UNESCO], 2017). Although many other communities are also leading ICBM, much of the ICBM literature in the OSR includes programs involving or led by these communities.

In 2011, the Teck Resources Limited applied to the Alberta Energy Regulators (AER) for approval of the Frontier Oil Sands Project. The project request included construction, operation, and reclamation of an existing oil sands surface mine located approximately 30 km south of Wood Buffalo National Park (AER & Canadian Environmental Assessment Agency, 2019). In response to the proposed Frontier project, the MCFN produced an IK and Use Report for Teck Resources that identified 357 site‐specific use values (i.e., cabins, burial sites, medicinal plants, critical habits for important species such as buffalo, moose, and caribou, hunting sites, berry‐gathering sites, and key transportation roots) within the Frontier project local study area, which is still widely used by community members (Candler et al., 2013, 2015). Many of these sites were within the Athabasca Delta, located in the lower southeast region of Wood Buffalo National Park. As proposed, the Frontier project would surely deepen existing impacts on land and resource use of value to MCFN members and consequently their IK and Section 35 Rights (Candler et al., 2013, 2015).

In 2014, to address potential impacts in Wood Buffalo National Park, the MCFN petitioned the World Heritage Committee to recognize the state of conservation and potential threats to the park and surrounding areas (UNESCO, 2017). The severity of environmental concerns detailed in the MCFN petition received enough support from other Indigenous communities, environmental organizations, and scientists to attract attention to the issue (UNESCO, 2017). Specifically, the MCFN identified a unique herd of bison, the Ronald Lake bison (*Bison bison athabasca*), specific to the region, as potentially the last remaining Wood bison unaffected by disease spread through the introduction, many years earlier, of Plains Bison *(Bison bison bison)* by authorities (Candler et al., 2015; MCFN, 2016b; Straka & Gray, 2018; UNESCO, 2017). Opportunity to include MCFN and IK in the decision‐making process involving bison introduction could have prevented the spread of disease among a culturally significant species. Call to Action 2 in Table 1 calls on natural scientists to recognize that understanding the landscape is a shared goal and, where possible, collaboration with Indigenous people can provide better outcomes (Wong et al., 2020). When knowledge is shared and developed collaboratively, management decisions are less likely to be dictated externally, and everyone can benefit from the process (Johnson et al., 2016; Spak, 2005; Thompson et al., 2020).

Responding to the petition in 2015, the World Heritage Committee requested that Parks Canada (Canadian Government) undertake a strategic environmental assessment (SEA) to assess the potential cumulative impacts of various developments, including oil sands, on Wood Buffalo Nation Park (World Heritage Site) and PAD. The completion of the SEA was a collaborative effort between governments, researchers, nongovernmental environmental organizations, and Indigenous communities in and around the Wood Buffalo National Park (MCFN, ACFN, Fort Chipewyan Métis Local 125, Little Red River Cree Nation, Salt River First Nation, Smith's Landing First Nation, K'atlodeeche First Nation, Deninu K'ue First Nation, and Northwest Territories Métis Nation). The SEA involved extensive information gathering including IK and WS, intended to inform project‐level environmental assessments and to influence the development of an action plan to protect Wood Buffalo National Park (Integrated Environmental Consultants, 2018). The efforts to protect the park subsequently led to the preparation of a Wood Buffalo National Park Action Plan intended to influence the design of environmental monitoring programs, as a collaborative approach to environmental problem‐solving that includes the participation of Indigenous peoples (Parks Canada, 2019).

In 2018, as part of Parks Canada's review for the proposed Frontier Oil Sands Mine, the SEA was submitted to the Joint Review Panel for consideration (Integrated Environmental Analytics, 2018). At that time, the MCFN also presented a letter to the Chair of Frontier Oil Sands Mine, identifying the necessity of assessing impacts on the exercise of Aboriginal and Treaty Rights (MCFN, 2018). In the letter, the MCFN and ACFN recommended a biodiversity stewardship area to permanently protect and support exercise of Treaty Rights between local wildlife, including the Ronald Lake bison (*Bison bison athabasca*; AER & Canada Environmental Assessment Agency, 2019). The letter draws on key principles that extend beyond environmental impact assessments, including perceived quality and quantity of resources, as well as land accessibility to know and teach Indigenous law and practices of environmental stewardship (MCFN, 2018). Call to Action 1 in Table 1, encourages understanding and respecting the sociopolitical aspects of the landscape (Wong et al., 2020). When assessing impacts on land, there is a strong need for natural scientists to understand community perspectives, what constitutes an impact, and how methods, indicators, and thresholds should be community driven and/or culturally appropriate (MCFN, 2018).

In response to the letter, Teck entered into participation agreements with MCFN and ACFN, requiring Teck to continue to consult with Indigenous groups on all plans required for the Frontier project. Included in the agreement, Teck was to support the MCFN and ACFN in the development of water‐quality monitoring programs and the installation of ambient‐air‐quality monitoring stations to address community concerns. Although Teck has funded water‐quality programs for the MCFN and ACFN, discussed later, it remains to be seen how they will achieve biodiversity objectives. This is because the project, if approved, would likely result in the loss of wetlands and old‐growth forests in the area (AER & Canada Environmental Assessment Agency, 2019).

The principles raised by the MCFN for the Frontier project, along with the 10 Calls to Action proposed by Wong et al. (2020; Table 1), are fundamentally vital in ICBM and can help Western scientists to meaningfully incorporate IK in environmental monitoring. Actions undertaken by the MCFN and ACFN to improve the involvement of Indigenous communities in environmental monitoring have greatly influenced the development of the OSM program governance structure. Both the MCFN and ACFN participated in the development of the OFA and are involved in many different ICBM programs discussed throughout this paper.

## ICBM CONCEPTUAL MODEL

Conceptual models are designed to be tools for organizing and communicating knowledge, and can offer space for mutual understanding among holders of traditional, local, and scientific knowledge (Suter, 2007). This ICBM conceptual model for oil sands environmental monitoring was developed through a WS lens. Indigenous communities were consulted through their participation in the governance of the OSM program. Owing to the timing for the development of a strategic plan for ICBM within the program by the ICBMAC, direct authorization and input from communities was not possible. Currently, many ICBM programs in the OSM program are in early stages of development and have yet to produce and report monitoring data contributing to the development of conceptual models. However, the monitoring program's commitment to adapt requires constant model revision as hypotheses are tested, including input from Indigenous communities. Furthermore, although the authors of the present paper recognize the inextricable link between environmental health and human health, human health is outside the scope of this environmental monitoring review. Through an Indigenous lens, this paper would be incomplete without the incorporation of human health and a holistic view of the ecosystem.

**Figure 2:**
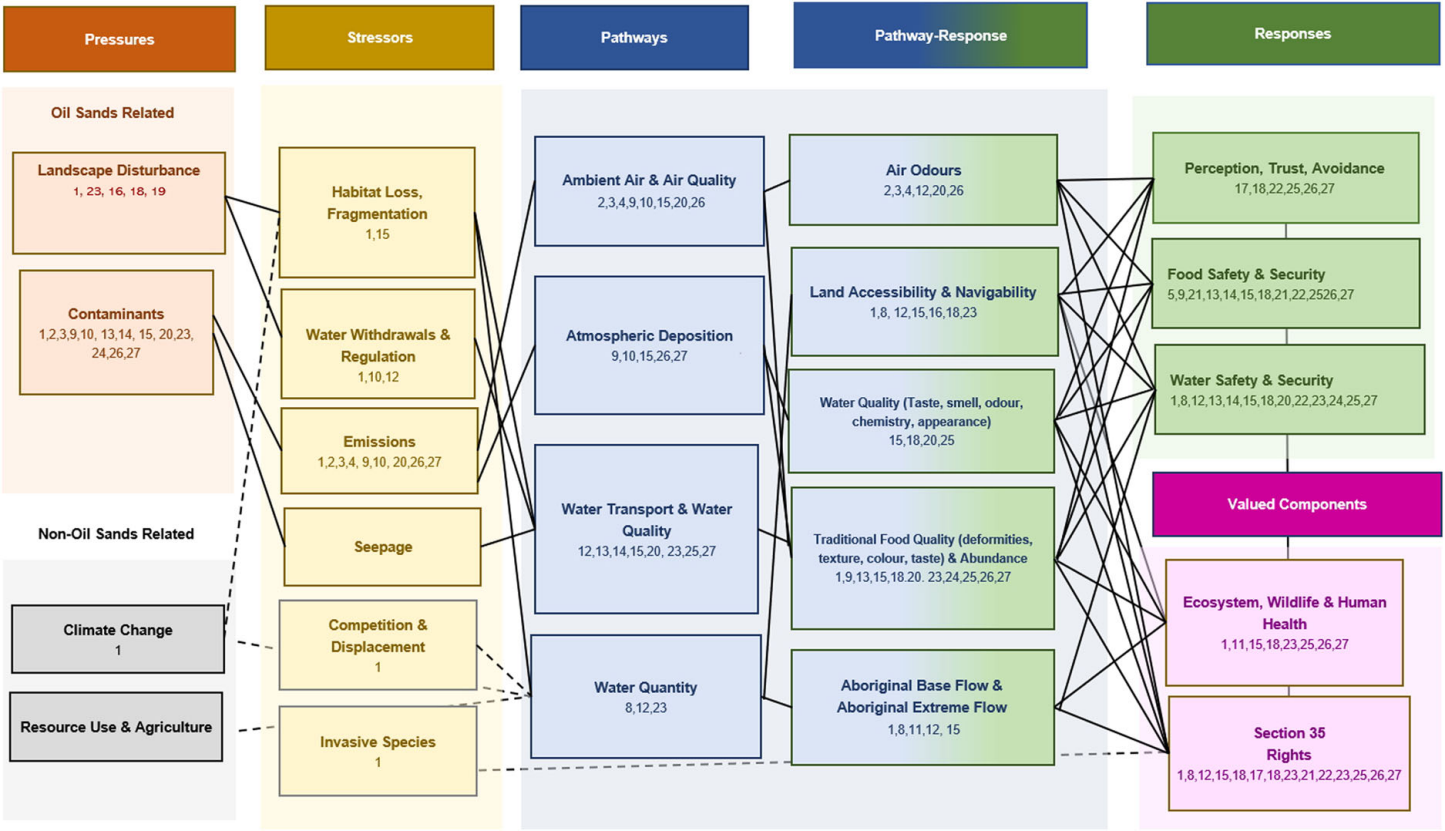
Conceptual model developed from a Western science lens to organize Indigenous community‐based environmental monitoring literature. The conceptual model is useful to determine where previous research has been focused and hypothesize potential relationships between environmental pressures and stressors, pathways, and responses. The dashed lines indicate a relationship between boxes; however, these relationships are not explored in this review

Overall, the scope of OSM programs is limited to impacts related to oil sand, which must be linked to a conceptual model and organized along a pressure/stressor‐pathway and response continuum (Roberts et al., 2021b). In this WS approach, the ICBM conceptual model, as well as the terrestrial model (Roberts et al., 2021a) focuses heavily on pathway‐response and response‐based changes (right side of the model). In the context of ICBM, pathway‐responses observed in the environment by Indigenous peoples include more complex components affected by change that relate to Section 35 Rights and cultural values (valued components). In the OSM program, some WS monitoring, such as air quality (Horb et al., 2021), focuses on stressor‐pathway relationships (left side of the model). These programs tend to focus on emissions and associated impacts on air quality (pressure/stressor‐pathway relationships). Some air programs study the impact of atmospheric deposition particles on terrestrial ecosystems. The conceptual model characterizes the inclusion of IK in these projects as responses, focusing on the heath of receptors (berries and vegetation) potentially exposed to deposition particles (stressor‐pathway). Some programs, such as surface water (Arciszewski et al., 2021), may align best as a pathway but can also be viewed as a receptor when assessing quality. Regardless, the integration of all multimedia and knowledge systems (organized through Figure 2) is necessary to determine what is changing in the environment, how it is changing, and why. The separation of indicators on the conceptual model is a visual tool to help address gaps in current monitoring programs. Optimally, monitoring programs would be designed to extend from one side of the model to the other and explore relationships and pathways between physical and chemical pressures and stressors with various ecosystem responses, including culturally relevant indicators and valued components.

The OSM program is a reputable source of information to policy with an objective to consider Canada's economic goals and protect the health of our Indigenous communities and environment. Hypotheses underpin the structure of the OSM program, representing possible ways by which pressures related to oil sands result in or contribute to environmental change. The pressure/stressor‐pathway‐response shown in the conceptual model can also be helpful in linking possible connections among different contamination sources and pathways in cumulative effect assessments (Roberts et al., 2021b). To support our research, the ICBM conceptual model is presented with numbers associated with a supplemental literature table (Table S1), identifying where ICBM research and literature have been focused and where we can improve our efforts. The sources in Table S1 can also be found in the reference section of this paper.

Environmental pressures in the OSR can be categorized into two main themes: (1) landscape disturbances (associated with pipelines, seismic lines, temporary roads, oil and gas wells, mining operations, dewatering, and steam injection); and (2) contamination (through emissions onto land and water through the atmosphere, deposition, and seepage). Changes to the land influence the environment through multiple stressors and pathways, resulting in multiple idiosyncratic effects and responses, terrestrially and aquatically, that continue to challenge mitigation efforts (Arciszewski et al., 2021; Roberts et al., 2021a). In this context, concerns for Indigenous communities in the OSR include access to land, water, and traditional territories (pathways) and the health of the ecosystem (responses) when influenced by various environmental stressors that ultimately challenge meaningful exercise of Section 35 Rights (valued components). Although climate change is listed on the conceptual model, the OSM program is not in a position to identify or separate climate change impacts from impacts related to oil sands. It is possible that impacts captured within the program and identified on the conceptual model are cumulatively affected and/or intensified by climate change. However, at this point, understanding the impacts of climate change is beyond the scope of the OSM program and therefore beyond the scope of this review.

## TYPES OF ICBM

ICBM has traditionally been developed for purposes of better understanding change on the landscape by Indigenous communities and driven by the ability to exercise Section 35 Rights. From this perspective, ICBM can provide both the framework and the tools to monitor land‐use change as it threatens traditional uses (Johnson et al., 2016). We can also view ICBM as a tool to measure contaminants, because many ICBM programs are developed from local concerns about the impacts of contaminants on traditional food and human health. In such circumstances, community members can participate in the collection of samples that are then analyzed in a laboratory. Many communities are also interested in species and biodiversity monitoring, which may not necessarily relate to either contaminants or landscape disturbance (pressures), but could also be influenced by both. Consequently, although not exclusively connecting species with biodiversity, many WS programs have formed from the concept of linking species and biodiversity management to wild food security and accessibility (Wong et al., 2020).

Monitoring programs can involve Indigenous communities as participants or develop programs in collaborative ways. There are several possible approaches to involving Indigenous communities in regional monitoring, depending on community capacity, which include participatory, collaborative, or Indigenous‐led program types (see Table 2 for type details). However, unless programs are led by communities, they should not be considered as true ICBM, but instead as WS programs that involve Indigenous people. In this paper, we view true ICBM as community‐led environmental monitoring programs that enable Indigenous communities to collect, analyze, and report on changes to their lands and waters related to the potential impacts of development. Although Indigenous‐led ICBM programs are more desirable for communities, collaborative, and/or participatory involvement of Indigenous people in WS monitoring, although not classified as ICBM, are still important to address community concerns. Regardless of the approach used to address Indigenous concerns, data collected and analyzed by researchers must be communicated to the communities in a timely, culturally appropriate manner. Call to Action 3 in Table 1 highlights the importance of coproducing results to improve understanding and management of resources by both researchers and the communities involved in the research (Wong et al., 2020).

**Table 2:** Types of environmental monitoring involving Indigenous communities (adapted from Danielson et al., 2009; Johnson et al., 2016; Kouril et al., 2016)

Types of Environmental monitoring involving Indigenous communities		
Type	Lead	Project details
Indigenous‐led	Indigenous communities	Monitoring is developed, implemented, analyzed, and reported on by one or more Indigenous communities, based on community priorities seeking to answer community concerns. The lead community may involve technicians or Western scientists to support technical aspects of the project, but the community itself is in charge of the design and implementation of the monitoring program(s).
Collaborative	Indigenous communities and Western scientists collaborate from start to finish. (sometimes scientists will lead)	Everyone contributes to the planning, design, implementation, analysis, and reporting on the research, but the community may not lead in the interpretation, validation, or reporting of the research. Collaborative projects are in some cases developed by community‐led initiatives and sometimes by WS‐led concerns. Projects usually offer opportunities for both participants to build capacity and share knowledge.
Participatory	Western scientists	The community supports the project, most often as volunteer data collectors in the field. The research questions, methods, and analyses are driven by Western science but still may address community concerns. There may be opportunities for communities to be trained in science‐based methods for potential autonomous ICBM at a later date.

Next, we review ICBM programs and characterize them as participatory, collaborative, or Indigenous‐led. For the purposes of this discussion, programs are organized by pathway (Figure 2), focusing on best practices that can help improve ICBM and the integration of ICBM into regional monitoring programs. In the present paper, we separate ICBM by pathway to best reflect the division of the pressure/stressor‐pathway‐response relationships in the conceptual model and to complement this special series (also organized by pathways and/or media). In order to improve our understanding of ICBM in the OSR, this method of organization is useful to highlight regional gaps in model design and data integration across monitoring programs. ICBM involves the management of multiple media and can produce data on many aspects of the environment holistically. Outside the context of this paper, ICBM programs must be considered holistically and cannot be separated by pathway (i.e., air, water), ignore major drivers like climate change, or exclude aspects of human heath in environmental assessment and monitoring programs.

## ENVIRONMENTAL CONSIDERATIONS OF ICBM

### Air quality

Emission of substances to air is a prominent pathway linking oil sands industrial activity to the environment and Indigenous communities in the OSR. Ambient air quality is an important factor in the overall environmental health and livability of a region. Air quality in the OSR is measured relative to guidelines established by the Government of Alberta's Ambient Air Quality Objectives regional specific air‐quality management frameworks, national air‐quality standards, and can also include odors reported by communities in the OSR (Alberta Energy Regulator et al., 2016). Acute air quality refers to the quality of air examined that may be harmful when exposure durations are minutes to hours. The conceptual model (Figure 2) hypothesizes the relationship between emissions (stressor) released into ambient air (pathway), which can influence air quality and the occurrence of unpleasant odors (pathway‐response). When communities in the region experience unpleasant odors, it can lead to negative perceptions of ecosystem health and influence the practice of Section 35 Rights (valued components; Dennis et al., 2015).

In 1985, an Air Quality Task Force was established by the Wood Buffalo Environmental Association (WBEA) in response to concerns raised by the Fort McKay First Nation (FMFN) related to oil sands development (Wood Buffalo Environmental Association [WBEA], 2020). The WBEA is a nonprofit, science‐based organization that monitors air quality and terrestrial environmental effects in the Regional Municipality of Wood Buffalo. In 2016, the WBEA began working with the Alberta government to provide independent ambient air monitoring for the OSM program. The WBEA is an important part of the OSM program (Baker, 2020; WBEA, 2020) that now operates collaboratively with FMFM, Fort McKay Métis Local, Fort McMurray Métis Local, health agencies, and oil sands industries.

The WBEA monitors the air 24 h a day and 365 days a year under two main air‐monitoring networks. The first, the Atmospheric Pollutant Active Monitoring Network, is a continuous ambient‐air‐monitoring network providing data for the public, industries, and local Indigenous communities. As part of the OSM program, the network has as an objective to understand impacts specific to oil sands emissions compared with other anthropogenic sources (WBEA, 2020). The air‐quality programs operating under the Atmospheric Pollutant Active Monitoring Network have been designed by the governments of Alberta and Canada, as well as the WBEA, and do not represent true examples of ICBM programs. However, the Acute Odour Monitoring and Emergency Response and the Community Odour Monitoring Program (COMP), discussed next, offer opportunities to address community concerns regarding odors and air quality. The second network, discussed later, is the Integrated Atmospheric Pollutant Deposition Monitoring Network, which focuses on dry deposition and specific atmospheric pollutants that could endanger the environment (WBEA, 2019b).

### Acute Odour Monitoring and Emergency Response (participatory)

The community of Fort McKay, located 58 km north of Fort McMurray, is surrounded by eight open‐pit and three in situ oil sands projects and is exposed to cumulative air emissions (AER, 2017). Odors are a prominent issue and have created air‐quality concerns for Indigenous communities in the OSR, some of whom experience odors as often as one day in every three (Dennis et al., 2015; WBEA, 2015a; Westman & Joly, 2019). Odors similar to rotten eggs, cat urine, body odor, sulfur, asphalt, tar, oil, and smoke are often accompanied by headaches, sore eyes, and throat and nose irritations (Dennis et al., 2015). Given the high level of concern, the communities in Fort McKay requested that AER prioritize emergency response procedures to address threatening air‐quality concerns, to protect the health and safety of the community when concentration levels are high (AER & Alberta Health, 2016). The Acute Odour Monitoring and Emergency Response Program launched in 2016, is a comprehensive and inclusive monitoring program in the community of Fort McKay, led by the Alberta government and funded by industry as part of the OSM program. The objective is to provide local communities, industries, and regulators with air‐quality data specific to the complaints of odors, which can be used to trigger emergency response actions (WBEA, 2020).

Of primary concern to the community of Fort McKay are short‐term elevated acute sulfur dioxide (SO₂) events, which can result in dangerous short‐term air quality, odors, and haze within the community. The AER received 172 air‐quality complaints from Fort McKay community members between January 2010 and December of 2014. Owing to complaints, aligning emergency response air‐quality exceedances and odor complaints became a priority to the AER (AER & Alberta Health, 2016). To address concerns, in July 2017, the Waskow Ocho Pimatiswin emergency response acute air‐quality station was installed and operationalized in the community of Fort McKay. At this station, ambient‐air data are collected every 5 min and compared with early warning emergency response thresholds for hydrogen sulfide (H₂S) and SO₂ to trigger emergency response actions in Fort McKay if exceedances occur (Dubé et al., 2016; WBEA, 2020). Creating a response protocol to odor complaints will help to improve transparency in identifying the source of the odors. If exceedances do occur, local provincial authorities are notified automatically (WBEA, 2020). However, communities in the region continue to be on a constant state of alert for potential air contamination events.

### Community Odour Monitoring Program (participatory)

The Community Odour Monitoring Program (COMP) was launched in February 2013, with the objective of understanding the relationships between odors experienced in the communities and oil sands development (Dann, 2013; WBEA, 2020). To ensure that monitoring done in the region is relevant to the concerns of community members, COMP involves community members in Fort McMurray and Fort McKay, as well as in surrounding areas. Community members participating in the COMP identify and monitor perceived odors in the community (WBEA, 2014, 2015a, 2016). To track results, the program offered training for volunteers to log odor complaints onto user‐friendly mobile and/or computer applications (Table 1, Call to Action 5). For each observation, participants report the date, beginning and end of the observation, spatial location, wind conditions, intensity, and type of odor they were experiencing (WBEA, 2014, 2015a, 2016). The data from the COMP are quality assured and quality controlled in a web‐based administrative panel. From there, the data are compared with data collected from WBEA's ambient‐air‐quality monitoring stations as part of the Atmospheric Pollutant Active Monitoring Network (WBEA, 2020).

In the first year of the program, more than 150 odor days (days with seven or more air odor complaints) were reported, growing to more than 270 the following year, peaking both years in summer (WBEA, 2014, 2015a). The most common odors reported were asphalt/tar, fuel/solvent, or burned/smoke (WBEA, 2014, 2015a). It is also important to note that odors cannot be attributed to a single source and increased odor reports generated over summer may be attributed to forest fires that had occurred during that time (Horb et al., 2021). Recent reports have identified total reduced sulfur (TRS) and total hydrocarbons (THC) to be most associated with odor (WBEA, 2019b, 2020). SO₂ concentrations are usually not as odorous; however, air analyses reveal that concentrations of SO₂ may indicate an industrial source (Adamache & Spink, 2012; WBEA, 2019b). In 2017–2018, four occurrences in Fort McMurray coincided with elevated SO₂ levels, which suggested odors could be associated with industrial plumes carrying odor‐causing compounds (WBEA, 2019b).

Although not classified as ICBM, opportunities to participate in WS projects such as COMP can provide opportunities for Indigenous communities to participate in monitoring activities and address concerns related to air quality. These experiences can build capacity within the community to develop Indigenous‐led programs involving air quality and odors. Increasing community involvement in regional air‐quality programs over time can increase the amount of air‐quality data in the OSR and in remote locations that may only be easily accessed by Indigenous community members who live there. Ensuring the credibility of multiple datasets will take careful attention and training in WS practices where applicable (i.e., SOPs, QA/QCs). WS programs involving Indigenous peoples can also be integrated with other ICBM programs in the OSM program. Improving our understanding of stressor/pressure‐pathway‐response relationships in the OSR will involve linking air‐quality data (pressure and stressors) with responses in the environment, such as the impact of contaminant deposition on food quality and the perception of food safety. For example, the Indigenous‐led Berry Harvesting project, part of WBEA's Integrated Atmospheric Pollutant Deposition Monitoring Network, discussed next, focuses on dry deposition and specific atmospheric pollutants that could endanger the environment and culturally significant resources (WBEA, 2020).

### Atmospheric deposition

WBEA's Integrated Atmospheric Pollutant Deposition Monitoring Network is designed to detect and quantify atmospheric pollutant deposition to the terrestrial environment, which includes impacts on traditional land and resources (WBEA, 2021). As shown on the conceptual model (Figure 2), emissions from oil sands industrial processes (stressors) are transported via the atmosphere and may occur as dry (e.g., dust) or wet (e.g., precipitation) deposition (pathways) on terrestrial and/or aquatic ecosystems (response). Deposited substances may be intercepted by vegetation such as culturally significant and/or medicinal plants and berries. These concerns have resulted in monitoring programs led by Indigenous communities to address this issue. In 2012, WBEA partnered with FMFN to develop the Berry Harvesting project, discussed next (WBEA, 2015b).

### Berry Harvesting (community‐led)

Indigenous communities living in the OSR have their own culturally relevant way of observing the effects (response) of emissions on the land because they have long‐standing IK that allows knowledge holders to recognize change (Foster et al., 2019). In 2010, Elders of the FMFN began noting changes in the quality and quantity of berries in traditional patches and approached the WBEA with their concerns (Baker, 2016, 2020; Foster et al., 2019). The Berry Harvesting project was consequently formed to explore contamination concerns (Oil Sands Monitoring Program, 2019). Berry‐harvesting sites were selected at varying distances from oil sands development, which included sites located close to open‐pit mines and one site located much further away. Elders provided IK about culturally significant blueberries and cranberries, including the state of the berry and berry flower, color, taste, abundance, and patch location (Baker, 2016; Parlee et al., 2012; WBEA, 2015b, 2019a, 2019b). Many IK holders expressed that they would not eat berries harvested from patches located closer to industries and would only eat berries from the site located farthest a way (Baker, 2016; WBEA, 2015b). This was determined using IK about the quality and quantity of the berries, comparing current observations with observations collected over generations.

Elders also requested that WBEA evaluate the concerns using the tools and approaches of WS. WBEA researchers examined contaminant and nutrient loads of berries that Elders harvested from each site. Additionally, ambient‐air‐monitoring stations were installed at each berry patch to record air quality and meteorological conditions (WBEA, 2019a, 2019b). Berries at each patch were analyzed for metals likely delivered via atmospheric deposition, including aluminum, iron, lead, vanadium, and chromium. Additional compounds associated with potential health benefits, including phenolics, anthocyanins, chlorogenic acid, and proanthocyanins, were also measured. In addition, at each berry patch, ambient‐air‐quality stations monitored SO₂, nitrogen dioxide (NO₂), and volatile organic compounds (VOCs), which can be emitted by both industrial and natural sources in the OSR. Chemical analysis suggested the concentration of phenolics, chlorogenic acid, and proanthocyanins were highest in berries collected farthest away, relative to all other locations. Analysis of trace elements from the surface of berries and air‐quality data revealed that berries further away from oil sands operations had lower monthly average levels of aluminum, iron, lead, vanadium, chromium, and SO₂, NO₂, and VOCs concentrations compared with sites closest to oil sand operations. It is important to note the association between health‐promoting elements and contaminant loads on berries and proximity to oil sands development. From a WS perspective, the concentrations of the measured parameters on the berries collected were not associated with levels expected to affect the overall health of blueberry or cranberry plants (WBEA, 2015b). However, proximity to oil sands activities can be associated with hesitancy of Indigenous peoples to consume berries.

Although many ICBM projects funded under the OSM program are in their infancy, the Berry Harvesting project has been ongoing for nearly 10 years and has only recently become a part of the OSM program. Representing best practices, the project offers a way of conducting collaboratively designed research to improve relationships between researchers and Indigenous communities from onset to completion (Table 1, Call to Action 10). Community members have the opportunity to review drafts of annual reports and decide on changes and approaches to monitoring, based on their own environmental observations, IK, and community needs (Baker, 2020). The project continues to evolve and has recently expanded to include four additional Indigenous communities in the Athabasca region (Fort McKay Métis, Fort McMurray First Nation, Fort McMurray Métis, and Conklin Métis). Communities are keen to continue building and growing their individual programs (WBEA, 2020). In years to come, we will be able to learn from the Berry Harvesting project and the FMFN in the design of ICBM nested within regional environmental monitoring programs. Using the conceptual model (Figure 2), the stressor‐response relationship between berry quality (i.e., traditional food quality) and atmospheric deposition, may help increase understanding of the state of the environment in the OSR and further our knowledge of atmospheric deposition patterns, impacts, and spatial proximity. With more data from ICBM over time and space, the OSM program will be better able to understand these relationships.

### Water quality

Changes in the quality of water are a concern of communities in the OSR. Concerns over water quality are most significant for communities living downstream of open‐pit mines and outside mining areas (Westman & Joly, 2019). Using the conceptual model (Figure 2), relationships potentially linking oil sands development and Indigenous community concerns over water quality include: (1) industrial water use, influencing water withdrawal and river flow (stressors); (2) land disturbance, influencing habitat quality and availability (stressor); and (3) contaminants (pressure) through industrial emissions (stressor) to the atmosphere (including snow melts containing emission particles) that may deposit as dust or precipitation into water bodies though deposition and/or dispersed by water transport and/or occur through industrial leaks and tailings ponds (pathways). Understanding the link between culturally identified indicators (such as smell and taste) and water chemistry can help connect responses observed in the environment in a stressor‐pathway‐response relationship (one side of the conceptual model to the other). When able, determining the cause of a response may mitigate changes. Developing capacity within communities to measure water quality can empower community members to increase security in resource consumption, including opportunities for knowledge sharing and transmission of IK to younger generations. Within the OSM program, there are both WS‐led and Indigenous‐led programs to monitor water quality, discussed next. Although Indigenous‐led programs are optimal for communities, both programs involve addressing concerns about the impacts on water quality in the OSR that are important to Indigenous communities.

### Wabasca Lake Monitoring Project (participatory)

The Wabasca Lake Monitoring Project was conducted in 2016 and 2017 by the Environmental Monitoring and Science Division of Alberta Environment and Parks, with participation from Bigstone Cree Nation, and is part of the province's ongoing Indigenous Lake Monitoring Program. The Indigenous Lake Monitoring Program is integrated with the WS‐led Provincial Lake Monitoring Program designed to collect baseline lake quality data across Alberta (Government of Alberta, 2019a, 2019b). As a pilot project, the Wabasca Lake Monitoring Project was one of the first OSM participatory lake monitoring programs undertaken in response to Indigenous community concerns about the quality of lakes of local importance and impacts on resources (Government of Alberta, 2019a, 2019b). Specifically, the Wabasca Lake Monitoring Project was initiated to meet Bigstone Cree First Nation's concerns about water quality and the health of walleye (*Sander vitreus*; a culturally significant fish) in Wabasca Lake. Community members were worried about safety of fish consumption owing to abnormalities observed in the fish, which included visible tumor‐like abrasions on fish skin. The goals of the project were to address gaps in water‐quality knowledge and information about North Wabasca Lake and to provide monitoring training in scientific sampling methodology to Bigstone Cree Nation (Zurawell et al., 2019).

The project provided opportunities for community members to learn to collect samples and water‐quality information. During the project, participants went out on boats with researchers to collect water samples (chemical, physical, and biological measures) during summer (June–September) and winter (February). Ten members from Bigstone Cree Nation participated in safety and sample collection workshops. Overall, the work revealed no metal contamination derived from human activity in the watershed. In addition, skin tumors on recently caught walleye that concerned community members were determined to be caused by a natural virus known as Lymphocystis, commonly found in Alberta's lakes. The virus is not usually fatal to fish and is not known to affect humans and other mammals (Zurawell et al., 2019). Although the project is not a true ICBM program, it allowed Bigstone Cree Nation to understand more about the health of fish that were of concern. The OSM program aims to continue the inclusion of Indigenous communities in monitoring lakes of local concern to address scientific knowledge gaps (Government of Alberta, 2019a, 2019b). Increasing local capacity to monitoring water quality where there are concerns can lead to improving security in local resources and improve the OSM's regional monitoring program by increasing the amount of data collected over time and space. However, to improve the effectiveness of the regional program, steps can be taken to include IK indicators of concern (appearance, taste of fish, and water) into WS‐based monitoring programs. It remains to be seen how the OSM program will develop and include culturally significant indicators in the Provincial Lake Monitoring Program.

### MCFN and ACFN water quality ICBM programs (community‐led)

Considerable research and water‐quality monitoring has been done to assess impacts of contaminants on lakes, rivers, sediment, and snowpack in the Alberta OSR. Of particular concern are polycyclic aromatic carbons (PACs). PACs represent a broad range of chemicals, including polycyclic aromatic hydrocarbons (PAHs). PAHs are organic hydrophobic compounds released from many sources in the Lower Athabasca River system, some naturally from oil sands deposits and others from extraction and upgrading facilities, forest fires, urban runoff, and industrial activities. Consequently, PAHs can be taken up by plants, accumulate in sediments, and can be found in tissues of terrestrial and aquatic organisms (Golzadeh et al., 2021; MCFN, 2015b, 2016a). Indigenous communities living around industrial facilities are concerned about exposure to PAHs through the consumption of traditional foods and water (Golzadeh et al., 2021). Concerns about water quality raised by the MCFN and the ACFN including potential impacts of PAHs are addressed by ongoing ICBM programs led by the MCFN and ACFN.

Among the first communities to lead water‐quality ICBM programs in the OSR, the MCFN began monitoring water in PAD in 2008. The PAD is one of the largest (3900 km^2^) and ecologically complex freshwater deltas in the world, filled with rivers, lakes, channels, marshes, and grasslands (Peace‐Athabasca‐Delta Environmental Monitoring Program, 2020) and is very significant to Indigenous communities in the region for water navigation and resources. ICBM developed by the ACFN and MCFN initially provided information about safe navigation in river channels, discussed later. However, in 2014, Teck Resources Limited, in an agreement with MCFN and ACFN, provided funding for the ICBM program to include monitoring of PAH levels in the Athabasca River, its tributaries, and the PAD (MCFN, 2016a). Designed to be operating only 30 km south of Wood Buffalo National Park, the Frontier project, if developed, might affect the park and the PAD's ecosystem through aerial deposition of particles containing contaminants such as PAHs (AER & Canadian Environmental Assessment Agency, 2019).

The objective of the ICBM program is to assess PAH signatures in water samples to determine if an oil sands influence could be detected downstream of industrial activities in the vicinity of Fort Chipewyan. Water samples collected by the community members nearest oil sands facilities contained concentrations of petroleum‐derived PAHs that were up to three times higher than background samples. This was recorded by the community as far as 50 km away from the center of development (MCFN, 2015a). Water‐quality sampling under the ICBM program suggest that, although sites at the mouth of the Athabasca River are more likely influenced by petroleum‐derived PAHs, reference sites are more likely influenced by forest fire activity and/or residential wood burning (MCFN, 2015a). Communities in the Fort Chipewyan area continue to observe floating patches of yellow‐brownish foam, rusty colored and foul‐smelling water, and scum left in pots after boiling. Some community members report they no longer drink the water from lakes or rivers as a result of these observed changes (Longley, 2015; MCFN, 2015b, 2016a).

Growing concerns about water quality can limit accessibility to exercise Section 35 Rights. Although the Canadian Council of Minister of the Environment (CCME) established guidelines to identify national water‐quality criteria for drinking water, communities may not agree with these thresholds (CCME, 2011). Currently, WS‐based surface‐water‐quality monitoring programs in the OSM program operate on benchmarks associated with CCME guidelines, in addition to 38 indicators under the surface‐water‐quality management framework for the Lower Athabasca Regional Plan (Arciszewski et al., 2021; Government of Alberta, 2017). However, it is possible that differences in understanding the quality of water and safety of resources can be supported by ICBM programs that use multiple, evidence‐based approaches to braid knowledge systems. Using the conceptual model (Figure 2), when assessing water quality, we can compare water chemistry with IK (i.e., pot scum, taste, smell) to examine possible relationships between the cause of observed changes along the pressure/stressor‐pathway response model. Although the OSM program may not be at capacity to integrate IK indicators into WS‐based water‐quality assessments, ICBM programs in the years to come can be designed to develop and integrate IK indicators in multiple evidence‐based approaches.

Overall, as a community‐led ICBM program, community members can be empowered to answer specific questions that are meaningful and important to them. Increasing capacity within communities to develop ICBM to monitor the quality of water can also provide security and access to traditional resources. ICBM can also improve transparency of data results and potentially contribute to OSM's regional surface‐water‐quality data repositories, including sampling in remote locations that may not be easily accessed by non‐community members. Currently, the MCFN place their water‐quality data on the Makenzie Data Stream (2021) database, which is openly accessible. The OSM Surface Water program currently uses KISTERS (2021) to house OSM data; however, it may not be recognized by communities or support community needs to store sensitive information. In the past, issues involving the collection, storage, accessibility, and usage of ICBM data, especially if the data contains sensitive information such as sacred IK, have been an issue for communities participating in regional programs (Dowdeswell et al., 2010). As part of OSM program work plans in 2019–2020, it remains to be seen how the ACFN and MCFN contribute WS‐based water‐quality data or how the OSM program will integrate ICBM and IK into the regional program. Regardless, utilizing data systems that are easily accessible for all users can offer new opportunities for data integration over time and space with greater involvement for communities to control, store, share, and protect information when participating in or developing regional environmental monitoring programs (Kanu et al., 2016).

### Fish and clam health monitoring

Using the conceptual model (Figure 2), pressure from emissions and industrial water use may influence water quality and quantity, and may directly and/or indirectly affect fish and other aquatic organisms through water transport, deposition and habitat disturbance (pathways). ICBM programs in the OSR have focused on ecosystems responses (pathway‐response) such as abnormalities in fish health (i.e., physical appearance, abundance, and behavior). Common physical abnormalities of poor fish health observed by community members include “skinny fish” (length‐weight ratio), deformities (tumors, deformed skulls, skeletons, and fins, pushed in faces, crooked tails, and bulging eyes), the quality of flesh (color, fat around the organs, taste, smell, and parasites), and fish texture (MCFN, 2015a, 2016a, 2016b; Parlee et al., 2012; Timoney & Lee, 2009). However, reasons for abnormalities in fish are not clear (Arciszewski et al.,  2021) and can influence local perceptions of fish health (response) and access to Section 35 Rights (valued components). Understanding why community members are observing changes in fish, based on their historical experiences, will require braiding IK and WS to link changes in fish with contaminant data or other stressors (parasites, temperature change, etc.). The OSM program supports fish monitoring programs, discussed next, developed either by or in collaboration with Indigenous communities in the OSR. Programs are designed to build capacity and enhance the relevance of environmental monitoring strategies by examining relationships between physical and chemical stressors and culturally relevant indicators.

### Whitefish Camp (Indigenous‐led, collaborative)

In 2018, the MCFN and ACFN expanded water‐quality monitoring activities to include monitoring of whitefish. Funded by the OSM program and Tracking Change (2016), MCFN and ACFN Elders, youth, and researchers attended Whitefish Camp. The camp was held at Lake Clair, located approximately 200 km north of Fort McMurray on traditional territory in Wood Buffalo National Park. During the camp, Elders shared IK and ways of life with youth (Tracking Change, 2016). Researchers were provided the opportunity to learn more about the concerns of the community and how they monitor the health of whitefish. The program was a success and, in 2019, the MCFN and the ACFN invited Smith's Landing First Nation to attend a Chip Fish Camp to spread the knowledge of fish sampling techniques and encourage knowledge‐sharing activities among knowledge holders. Table 1, Call to Action 4, calls on researchers studying wildlife to seek advice from Elders for respectful ways of handling that wildlife, including specific methods and protocols that communities may use to demonstrate their stewardship for the land that researchers should also follow (Whyte et al., 2016). These examples have been seen in collaborative fish harvesting, where fish are jointly prepared by both researchers and community members in accordance with local customs and values (Wong et al., 2020). Samples of whitefish were collected in Lake Claire and analyzed in a laboratory to measure levels of PAHs, total mercury, and isotopes. Data collected will build on existing data collected by MCFN and ACFN. At the Fort Chip Fish Camp, participants engaged in traditional methods of drying the healthy fish, and knowledge holders connected to share stories, knowledge, and experiences with each other (Jones, 2019).

Lack of clarity regarding changes in whitefish abundance or quality can influence a community's ability to practice Section 35 Rights. Do the changes observed in whitefish mean they are not safe to eat? Answering this question requires an improved understanding of contaminant and other stressor‐pathways in relation to the health of whitefish, potentially affected by development activities. A multiple evidence‐based approach, in which IK (i.e., changes observed in whitefish by community members) is braided with WS (i.e., contaminant data), can help improve understanding of whitefish health and provide communities the ability to monitor culturally significant resources. Braiding IK and WS regarding the health of fish can address the OSM program's objective to document change and potentially begin to link change to specific development activities, such as oil sands. Using the conceptual model (Figure 2), changes captured in fish (IK, physical or chemical responses) can be linked with pathways of exposure, such as water quality and quantity, which can then be linked to potential sources (pressures and stressors). Where change is hard to capture or is potentially influenced by multiple pressures and stressors and pathways, incorporating IK where there is greater variation in WS can strengthen hypotheses for contamination pathways of aquatic species. Although the OSM program may not be at capacity to integrate IK indicators into regional fish health assessments, ICBM in the years to come can be designed to develop integration strategies for IK indicators (such as fish texture, color, taste) in multiple evidence‐based monitoring approaches that are either WS‐based or Indigenous led.

### Freshwater Clam project (Indigenous‐led)

Indigenous communities in the northeastern oil sands region, in particular the Fort McMurray Métis, are concerned about the disappearance of freshwater clams in the Athabasca River. IK holders in the region reported that freshwater clams have declined and, in some areas of the Lower Athabasca, have completely disappeared over the past 20–40 years. The Fort McMurray Métis rely on and are intimately connected to these waterways for navigational purposes and for traditional resources, which for generations has included harvesting freshwater clams (Hopkins et al., 2019). In response to these concerns, in 2017, the Fort McMurray Métis and the governments of Canada and Alberta initiated the Freshwater Clams Project to understand why the freshwater clams were disappearing. The Freshwater Clam Project is a collaborative ICBM program led by the Fort McMurray Métis grounded in Métis IK and methodology, which guides the program in a culturally meaningful way (Government of Alberta, 2017).

In 2018, after the program's first year, the project expanded to include a larger sampling area to improve density and distribution of freshwater clam populations and increase sample sizes of clams, water, and sand sediments along the Athabasca and Clearwater rivers (Government of Alberta, 2018). The Fort McMurray Métis directed site selection in the Clearwater and Athabasca rivers to collect data and identify impacts from industrial activities, including oil sands industries and agricultural influences. Data recorded at each site included the time, dates, and weather conditions of past and present land uses, as well as descriptions of changes to freshwater clams, vegetation, water, and riverbed characteristics. To build on IK, the project also used WS to explore potential factors that could influence freshwater clam health, which included chemical analyses in clam tissue, sediment, and water samples analyzed by accredited laboratories. During field exercises, videos were taken and livestreams were shared with Fort McMurray Métis in real time (Hopkins et al., 2019).

The Clam Project demonstrates many of the Calls to Action shown in Table 1, and is a good example of how to braid IK and WS. The project successfully facilitates partnerships and creates safe spaces to address questions about freshwater clam health in culturally relevant ways. Throughout the project participants engaged with the land, using local river navigation, harvesting protocols, and ceremonial practices (Hopkins et al., 2019). Hopkins et al. (2019) highlights the importance of knowledge translation through community workshops, presentations, laypersons reports, government reports, and scientific reports, as well as opportunities for Elder–youth knowledge exchange. The use of IK to determine clam health is identified through culturally relevant indicators to shape what is measured, where, and when. Braiding knowledge systems in this ICBM program was effective in building trust and mutual respect among participants, while they learned from each other how to answer important research questions.

### Water quantity

Research suggests that discharge into the Athabasca River has declined over the past 50 years (ACFN, 2019; Candler et al., 2010, 2015; Carver & MacLean, 2016; Lawe et al., 2005; MCFN, 2016b; Parlee et al., 2012; Straka & Gray, 2018; Westman & Joly, 2019). Flow of the river can be influenced by many factors including the flow rate of the Peace River as it merges with the Athabasca River, water withdrawals related to oil sands, and the development of the W.A.C. Bennett Dam, located at the mouth of the Peace River in Hope, British Columbia (Candler et al., 2010; Carver & MacLean, 2016; Timoney & Lee, 2009). Using the conceptual model (Figure 2), various environmental stressors (industrial water use, land disturbance, climate change) can create insufficiencies in water quantity (pathway) causing changes to levels and/or flow rates of rivers and tributaries (responses) throughout the PAD. These changes can ultimately influence access to traditional territories and limit the practice of Section 35 Rights (valued components). Change in flow and depth of water in the PAD can also influence the survivability of semi‐aquatic wildlife, such as culturally significant muskrats (*Ondatra zibethicus*; MCFN, 2016b; Straka & Gray, 2018). Although changes in water quantity are not the only cause for concern (i.e., changes to landscapes and ecosystems, loss of habitat, species abundance), it has been a primary focus of ICBM led by the MCFN and ACFN, discussed next.

### Aboriginal base flow (community‐led)

To address declining water levels in the PAD limiting access to traditional territories, in 2011, the ACFN and MCFN initiated an ICBM program to track impacts on the PAD. Driven by community concerns regarding access loss, research questions shaped the design of the study around land accessibility in a culturally meaningful way. The objective of the monitoring program is to quantify the extent of traditional territory access loss over time and space (Carver & MacLean, 2016; Tracking Change, 2016). Understanding how access points correlate with discharge of the Athabasca River and the level of water in the PAD may lead to understanding more about thresholds for traditional use (Carver & MacLean, 2016). Drawing on IK, the ACFN and MCFN defined thresholds (aboriginal base flow and aboriginal extreme flow) required for navigability in the PAD (Candler et al., 2015). The aboriginal base flow is defined as the level of flow required below which widespread disruption of traditional use can likely occur. Aboriginal extreme flow is defined as the discharge flow required by the Athabasca River above which levels are unlikely to cause impediment to the ability of ACFN and MCFN members to practice their rights and access harvesting areas (Carver & MacLean, 2016).

Through the ICBM program, Guardians monitor 12 sites along the Athabasca River and in the PAD (Tracking Change, 2016). As part of the ICBM program, monitoring sites highlight key pinch points, selected based on important observations from community members collected over the past 20 years. In this context, pinch points represent water passageways essential to accessing territory that are the first to become impassable as water levels decline. While monitoring the sites, Guardians record the GPS position of pinch points, collect photos of the sites, record general weather conditions, air temperature, barometric pressure, wind direction and speed, water depth, water flow direction, and water temperature. After five years of data collection, regardless of variabilities in depths of pinch points, strength of correlations (varying by location), and extent of river dynamics, the water depth at each site persistently converges when the flow of the river drops below approximately 500–600 m^3^/s. This means that if the flow of the Athabasca River decreases below 500–600m^3^/s the low flow may lead to widespread loss of the ability to navigate waterways to access traditional territories. The concern is that the effects of oil sands withdrawals from the Athabasca River during critical use periods (typically during low water seasons) can disproportionately influence navigability than if the river had higher discharge volumes (Carver & MacLean, 2016). To address these concerns, communities are asking for limitations to water withdrawals in the Athabasca, especially during low times and critical use periods, to improve security in traditional navigational pathways.

In their letter to the chair of Frontier Oils Sands Mine Frontier, referred to at the beginning of this paper, the MCFN identify that meaningful practices in ICBM involve recognizing the necessity of assessing impacts on the exercise of Aboriginal and Treaty Rights, including how approved activities affect this ability (MCFN, 2018). This ICBM program assessing water quantity is designed around these practices and represents a good example of true ICBM. Although it has yet to be seen how the data collected in this project will be integrated into the regional OSM program, the Aboriginal Base Flow and Aboriginal Extreme Flow can provide new opportunities for establishing guidelines for water withdrawal and dam regulators in ways that are meaningful to communities.

The MCFN and ACFN now collect data using a custom app and database system that is designed to secure and store data and which is compatible with other land‐use planning and regulatory management software (Indigenous Guardians Toolkit, 2021). As a source for navigation in the PAD, community members have access to the app to track and observe hazardous areas that may not be safe or navigable. Although this improves navigation safety, it may not lead to the provision of access. Overall, although water withdrawals related to oil sands and dam regulation can add stress to the PAD, especially during times of low water, water quantity decline has been a result of cumulative pressures over decades (Arciszewski et al., 2021). More time and information is required to improve our understanding of the impacts related to oil sands relative to cumulative effects over time and space. As the OSM program works to develop a cumulative effects program, work undertaken by the MCFN and ACFN will be important to include.

### Terrestrial and semi‐terrestrial wildlife and vegetation (multiple pathways)

Using the conceptual model (Figure 2), environmental pressures on the landscape can be categorized into two main themes: (1) landscape disturbances (pressures), associated with various stressors such as habitat loss and fragmentation (including seismic lines, roads, construction, and operations, etc.), noise, water withdrawal (dewatering), and regulation; and (2) contamination (pressures), associated with emissions (stressors) onto land and water through air, atmosphere deposition, industrial leaks, and seepage (pathways). Influenced by multiple stressors and pathways, changes to landscapes result in multiple idiosyncratic responses in terrestrial and semi‐terrestrial wildlife that continue to challenge mitigation efforts (Roberts et al., 2021a). Some terrestrial monitoring programs in the OSM program that involve the participation of Indigenous communities were developed from concerns about the impacts of contaminants on traditional food and human health, discussed next. Some communities have expressed interest in monitoring species abundance and biodiversity, which can be associated with contaminants, landscape disturbance, or a combination of both, discussed later. Regardless of the pathways or responses being captured in these programs, the objectives are driven by environmental stressors that ultimately challenge meaningful exercise of Section 35 Rights (valued components).

### Wildlife Contaminants and Toxicology program (participatory)

The Wildlife Contaminants and Toxicology program was initiated by scientists from the Environment and Climate Change Canada's Ecotoxicology and Wildlife Health Division, with additional funding from the OSM program. In collaboration with the MCFN and ACFN, the objective of the program was to study the impact of pollutants on wildlife health in Fort Chipewyan and surrounding areas. Designed to address the possibilities of multiple contaminant pathways, the Wildlife Contaminants and Toxicology program hosted workshops to discuss with community members what the animals were eating, what they were drinking, and where they were living. During workshops, members of MCFN and ACFN shared IK with scientists and youth about the wildlife in the area, such as which animal species should be monitored, where the monitoring should occur, and when. This information was vital to the success of the project and could not have been possible without the IK of community members who participated (Government of Canada, 2019).

As part of the program, community members collected wildlife (including muskrat, river otter, and waterfowl) from traditional territories and traplines. Tissue samples were sent for lab analyses to assess contaminant burdens. Semi‐aquatic species were chosen because they are closely related to water and are good indicators of ecosystem health. With a diet consisting predominately of fish, semi‐aquatic wildlife may be exposed to contaminants in the water and might accumulate toxins in muscle and organ tissues, especially the liver. The selection of semi‐aquatic species was made by scientists; however, which species, where, and when was guided by community members. The lab analyses of collected samples from participants indicated that wildlife collected closer to Fort McMurray industrial activities had higher levels of contaminants than wildlife collected farther away (Government of Canada, 2019). However, from a WS perspective, these levels are still safe for consumption. It is unknown how much community members trust these analyses; however, collaborative programs that build lasting relationships between Indigenous peoples and research scientists can improve trust in environmental monitoring data and management strategies in years to come.

Using the conceptual model (Figure 2), understanding the impact of contaminants (pressure) on wildlife requires the knowledge and participation of communities to address concerns related to food security (valued components) in the OSR. Overall, the Wildlife Contaminants and Toxicology program utilized the expertise of the MCFN and ACFN to guide the what, when, and where of monitoring design. The program also offered space for community members to share knowledge, build capacity, and learn about contamination, sampling, and dissection. Through these collaborative efforts, communities participating in this program are able to develop their own ICBM programs to assess impacts of contaminants on wildlife using both WS and IK (Government of Canada, 2019).

### Wetland Ecosystem Monitoring program (participatory)

The Athabasca and Peace oil sands regions of Alberta contain culturally significant wetlands valued for the quality and quantity of traditional plants. Culturally valued wetlands represent nearly half of pre‐disturbed landscapes in the Athabasca region (Garibaldi & Straker, 2009). UNESCO (2017) identified some regions of Wood Buffalo National Park as having unique and biologically diverse plant and wetland plant communities, which support some of the last remaining breeding grounds of the endangered whooping crane (*Grus americana*) and Wood Bison (*Bison bison athabasca*). The cultural importance of wetland plants can include many things such as food resources, medicinal products, and support for spiritual values. However, the IK acquired to discover these uses is a result of accessible, abundant, and biologically diverse wetland ecosystems over generations (O'Flaherty & Davidson‐Hunt, 2008). Using the conceptual model (Figure 2), some pathways to which pressures and stressors are known to affect wetlands include atmospheric deposition, water regulation, water withdrawals, and landscape disturbance. As a complex ecosystem, wetlands require collaboration across all monitoring theme areas (air, water, land) and knowledge systems to accurately assess wetland ecosystem health. A good example of this is the Wetland Ecosystem Monitoring program.

The Wetland Ecosystem Monitoring program is new to the OSM program. Launched in 2018, it was designed to detect and report change related to oil sands industries in wetland ecosystems. The program assesses three major pathways and observable effects in wetlands including: (1) atmospheric deposition (pathway) and the development of indicators to detect deposition from oil sands emissions (responses), (2) effects of hydrological alteration (pathway) and the development of indicators to detect alteration patterns (responses), and (3) impacts of land disturbance (stressor) and the development of indicators to detect changes on wetlands (responses). Each area involves the possibility for integration with other OSM programs focusing specifically on those theme areas. The primary goal of this program is to develop a long‐term wetland monitoring program that includes and applies IK related to wetlands and ecosystem change. As part of this goal, the program is integrated with the Culturally Important Wetlands Plants program. The objective of the Culturally Important Wetlands Plants program is to provide space and resources for communities in the OSR to share IK and develop ICBM programs that are culturally meaningful (Government of Alberta, 2019a, 2019b).

After the Culturally Important Wetlands Plants program moves past the pilot phases, monitoring health and abundance of indicator (culturally significant) species in wetlands can improve understanding of impacts associated with contaminants, landscape disturbance, or a combination of both on wetland ecosystems, which ultimately challenge the meaningful exercise of Section 35 Rights (Government of Alberta, 2019a, 2019b). Integration of stressor‐pathway focused programs (atmospheric deposition or hydrological alteration) with associated indicators species (responses) is essential to understanding the impacts of contamination and land disturbance on wetlands ecosystems. Together, relationships can be linked across the conceptual model (Figure 2). Synthesizing wetlands literature details the high level of integration of multiple programs and data. Owing to the infancy of the Wetland Ecosystem Monitoring programs, no reports are publicly accessible. All information in this section was obtained from OSM program work plans available online from the program website (http://environmentalmonitoring.alberta.ca). It remains to be seen how the OSM program will move these objectives forwards; however, the development of the programs is a good indication the OSM program is heading towards an integrated strategy that combines the strengths of IK and WS to tackle challenging environmental issues.

## DISCUSSION

Changes observed in the air, water, or land (including wildlife and vegetation) can alter behavior of Indigenous peoples because of concerns over the access and safety of traditional resources from these environments. This could prevent some community members from exercising their Section 35 Rights and passing IK on to the next generations. To participate, collaborate, or develop ICBM programs, Indigenous participants must be able to practice Section 35 Rights, experientially associated with the acquisition of IK. Therefore, true representations of ICBM are Indigenous‐led and deeply rooted in IK and language, cultural values, law, and practices of environmental stewardship associated with the meaningful exercise of Section 35 Rights. In this context, ICBM is not simply monitoring what remains, but also restoring IK through the resurgence of accessible traditional and cultural practices. When assessing impacts on land, there is an equal need for natural scientists to understand community perspectives, methods, indicators, and thresholds and for communities to understand WS methods of data collection and analysis. This report highlights key principles to meaningfully guide collaboration between researchers and community members, including the 10 Calls to Action developed by Wong et al. (2020) and referenced throughout this paper.

In reviewing best practices and principles of existing ICBM and WS programs involving Indigenous peoples in the OSR, focusing on the period 2009–2020, we discuss the importance of ICBM and WS‐based programs involving Indigenous communities in the OSR. Although the idea of integrating IK and WS to tackle challenging environmental problems is not new, the paper provides a unique way of organizing the integration of IK and WS using a conceptual model and a governance structure to support these collaborative efforts. It is not the intention of the present paper to adapt IK or WS to any model or monitoring program, but to discuss the integrative ability of both to better understand pressure/stressor‐pathways and response relationships of environmental impacts in the OSR. Contextualizing each program into the conceptual model is useful for large monitoring programs, like the OSM program, to integrate large amounts of data assessing different aspects of the environment over time and space. Using a conceptual model can enable regional monitoring programs to constantly evaluate research objectives and identify gaps in a collaborative way. As with the OSM program, the conceptual model is meant to be adaptable and inclusive of all knowledge systems and can be interpreted in many ways. Regardless of interpretation, the conceptual model is not complete without the application of both IK and WS.

Direct input from communities would have been valuable for this paper; however, other priorities prevented participation beyond internal governance structure. Communities may have their own ways of presenting relationships in the conceptual model; however, the presentation of relationships in this paper still offers the opportunity to examine the possibilities of data integration (WS and IK) to answer complex environmental questions. It is important to note that communities participating in the OSM program focus on reports generated for community use rather than traditional WS outputs. Although this is important for communities, it has limited the availability of reports and peer‐reviewed publications from ICBM initiatives for this review. As a result, a significant amount of the literature included in the review is gray literature or reports collected from communities, organizations, and government websites. As programs mature, more ICBM reports can be made available to the public and for OSM program use.

Currently, many ICBM and WS science programs involving Indigenous peoples in the OSM are still in their infancy. Best practices of ICBM were largely represented in ICBM programs led by MCFN and ACFN, most which were initiated before the formation of the OSM program. As leaders in ICBM, the MCFN and ACFN play a large role in the OSM program. ICBM initiatives by the ACFN and MCFN, although holistic in practice, focus predominantly on the impacts of contaminants on air and water. Currently, there is less documentation of land (including wetlands) and terrestrial wildlife monitoring. However, this does not characterize land and wildlife as less significant, but suggests that ICBM approaches currently support regional monitoring efforts where data are more easily captured in regional‐scale models, such as air and water quality. Data repositories and data mapping tools sensitive to the collection of various IK (i.e., vegetation, wildlife health, and abundance) are gaps in the OSM program that require attention. Valuable integration opportunities for ICBM exist in the OSM program, specifically with respect to aligning or validating geospatial products with community‐based observations of near‐real‐time environmental change.

Overall, from a regional perspective, communities can benefit from working with other Indigenous communities and scientists to share knowledge and data when participating in monitoring programs. Increasing the scope (area) of data collection can help address issues of incorporating local data into regional‐scale models. Using this strategy, communities can discuss what they want to know (research questions), what will be monitored (when and where), and how strategies integrating IK and WS can improve environmental monitoring over a large area. Regional‐scale data can improve the power of ICBM data in regional monitoring programs, advancing the ability to detect the likelihood of environmental impacts over time and space. In all capacities, the involvement of Indigenous peoples in the OSM program has moved the program closer to achieving this goal. Opportunities exist to increase data collection over time and space and, potentially, in areas not easily accessible by non‐Indigenous peoples. This can increase the potential for regional monitoring programs to address community concerns and also fill relational gaps visually guided through the use of conceptual models. When developed appropriately, ICBM programs can enhance the effectiveness and relevancy of environmental monitoring linking physical and chemical stressors (land disturbance and contamination) with culturally significant indicators (pathway‐responses and valued components) and address community concerns.

## CONFLICT OF INTEREST

The authors declare no conflicts of interest.

## DISCLAIMER

This article is written through a Western science lens, and the knowledge from, and perspectives of, Indigenous communities are largely absent. Communities were consulted through the governance of the OSM program; however, the development of ICBM framework through the ICBM advisory committee was a priority during this time. Information from Indigenous participants undertaking, collaborating, and/or participating in Indigenous community‐based monitoring programs are important components of the development of ICBM initiatives. Although the authors of this paper recognize the inextricable link between environmental health and human health, human health was outside the scope of this environmental monitoring review. Through an Indigenous lens, this framework is incomplete without the addition of human‐health considerations.

## SUPPORTING INFORMATION

Supplemental Table S1 is associated with a conceptual model in the paper (Figure 2), which organizes literature along a stressor‐pathway‐response model.

## Supporting information

This article includes online‐only Supporting Information.

Supporting information.

## Data Availability

No new data were generated by this literature review.

## References

[ieam4485-bib-0001] Arciszewski, T. J. , Ussery, E. J. , Hazewinkel, R. O. , & Dubé, M. G. (2021). A critical review of status of lakes and rivers from Canada's oil sands region. *Integrated Environmental Assessment and Management*, 18(2), 361–387. 10.1002/ieam.4524 PMC9298303 34546629

[ieam4485-bib-0008] Athabasca Chipewyan First Nation (ACFN) . (2019). *Dene‐lands and resource management*. Final Report on ACFN Community‐based Monitoring Program. www.acfn.com/acfn‐irc

[ieam4485-bib-0009] Adamache, L. , & Spink, D. (2012). *Cumulative effects: Concerns of Fort McKay regarding the impacts of emissions to air from industrial development*. Prepared for the Fort McKay Sustainability Department. https://landuse.alberta.ca/Forms%20and%20Applications/FMFN%20‐%20Application%20App%204%20Cumulative%20Effects_2014‐03‐05_PUBLIC.pdf

[ieam4485-bib-0010] Alberta Energy Regulator (AER) . (2017). *Improving air quality and odours in Fort McKay*. https://www.aer.ca/protecting‐what‐matters/reporting‐on‐our‐progress/improving‐air‐quality‐and‐odours‐in‐fort‐mckay

[ieam4485-bib-0011] Alberta Energy Regulator (AER), & Alberta Health . (2016). Dubé, M., Spangelo, J., Dumanski, M., Zelensky, M., Gee, H., Semaine, Z., MacDonald, C., Nixon, S., Saunders, B., Campbell, D., Wei, T., Falstead, L., Etris, P., Curran, B. (eds.). Recurrent Human Health Complaints Technical Information Synthesis: Fort McKay Area. https://www.aer.ca/protecting‐what‐matters/reporting‐on‐our‐progress/reports/recurrent‐human‐health‐complaint‐reports

[ieam4485-bib-0012] Alberta Energy Regulator (AER), & Canadian Environmental Assessment Agency . (2019). *Report of the Joint Review Panel: Teck Resources Limited Frontier Oil Sands Mine Project*. https://static.aer.ca/prd/documents/decisions/2019/2019ABAER008.pdf

[ieam4485-bib-0014] Baker, J. (2016). Research as reciprocity: Northern Cree community‐based and community‐engaged research on wild food contamination in Alberta's oil sands region. Engaging with Indigenous Communities, (2), 1. 10.15402/esj.v2i1.201

[ieam4485-bib-0015] Baker, J. M. (2020). Do berries listen? Berries as indicators, ancestors and agents in Canada's oil sands region. Ethnos, 86(2), 273–294. 10.1080/0014844.2020.1765829

[ieam4485-bib-0017] Bill, L. , Crozier, J. , & Surrendi, D. (1996). *A report of wisdom synthesized from the traditional knowledge component studies*. Northern River Basins Study. Edmonton, Alberta. http://www.barbau.ca/sites/www.barbau.ca/files/0‐662‐24768‐X_0.pdf

[ieam4485-bib-0019] Candler, C. , Gibson, G. , & Mikisew Cree First Nation (MCFN) . (2015). Wîyôw'tam'kitaskino (Our Land is Rich): A Mikisew Cree culture & rights assessment for the proposed Teck Frontier project update. Prepared for the [MCFN] Mikisew Cree First Nation. https://open.alberta.ca/dataset/5da3a4f0‐f982‐4f8e‐af9b‐cb00c39fb165/resource/360a4892‐0a07‐4388‐b7a2‐7ce6c2908cc9/download/mcfn‐wiyowtankitaskinofinal‐for‐pdfsept16.pdf

[ieam4485-bib-0020] Candler, C. , Olson, R. , DeRoy, S. , Athabasca Chipewyan First Nation (ACFN), & Mikisew Cree Nation (MCFN) . (2010). *As long as the rivers flow: Athabasca River knowledge, use and change*. Edmonton, Alberta: Park Institute.

[ieam4485-bib-0021] Candler, C. , Olson, R. , & The Firelight Group Research Cooperative . (2013). *Mikisew Cree first nation Indigenous knowledge and use report and assessment for Teck Resources Limited's Proposed Frontier Oil Sands Mine Project*. https://aeic‐iaac.gc.ca/050/documents/p65505/114463E.pdf

[ieam4485-bib-0022] Candler, C. , Olson, R. , & The Firelight Group Research Cooperative . (2015). *Addendum to Mikisew Cree First Nation Indigenous knowledge and use report and assessment for Teck Resources Limited's Proposed Frontier Oil Sands Mine Project*. https://ceaa‐acee.gc.ca/050/evaluations/document/114502?&wbdisable=true&culture=fr‐CA

[ieam4485-bib-0023] Carver, M. , & MacLean, B. (2016). Community‐based water‐depth monitoring in the Peace‐Athabasca Delta: Insights and evaluation. Prepared for the [ACFN] Athabasca Chipewyan First Nation and [MCFN] Mikisew Cree First Nation. https://drive.google.com/file/d/0B9zfvUxjd3bnZV9ROWZlYkliVnM/view

[ieam4485-bib-0024] Canadian Council of Ministers and the Environment (CCME) . (2011). *Protocol Manual for Water Quality Sampling in Canada*. https://ccme.ca/en/res/protocolsdocument_e_‐final1.0correctedisbn‐secure.pdf

[ieam4485-bib-0026] Corntassel, J. , & Hardbarger, T. (2019). Educate to perpetuate: Land‐based pedagogies and community resurgence. International Review of Education, 65(1), 87–116. 10.1007/s11159-018-9759-1

[ieam4485-bib-0027] Danielson, F. , Burgess, N. D. , Balmford, A. , Donald, P. F. , Funder, M. , Jones, J. P. G. , Alviola, P. , Balete, D. S. , Blomley, T. , Brashares, J. , Child, B. , Enghogg, M. , Flejdsa, J. , Holt, S. , Hüberts, H. , Jenson, A. E. , Jenson, P. M. , Massao, J. , Mendoza, M. M. , … Yonten, D. (2009). Local participation in natural resource monitoring: A characterization of approaches. Conservation Biology, 1(23), 31–42. 10.1111/j.1523-1739.2008.0163.x 18798859

[ieam4485-bib-0028] Dann, T. (2013). *Integration of odour data for the [HEMP] Human Exposure Monitoring Program*. RS Environmental, Ottawa, Canada. Prepared for [WBEA] Wood Buffalo Environmental Association.

[ieam4485-bib-0029] Dennis, J. H. , Spink, D. , Abel, R. , Stuckless, D. , & Fort McKay First Nation . (2015). *Alberta Oil Sands development and odour issues: The First Nation of Fort McKay's experience, perspectives and initiatives*. from: https://pdfs.semanticscholar.org/07a9/d7c856531de2b7c7cbdc1125526409658b19.pdf

[ieam4485-bib-0030] Dockery, A. M. (2012). Do traditional culture and identity promote the well‐being of indigenous Australians? Evidence from the 2008 NATSISS. In B. Hunter & N. Biddle, (Eds.), *Proceedings of the Social Science Perspectives on the 2008 National and Aboriginal Torres Strait Islander Social Survey Conference* (pp. 281–305), 11–12 April 2011. Canberra, Australia: The Australian National University.

[ieam4485-bib-0031] Dowdeswell, L. , Dillon, P. , Ghoshal, S. , Maill, A. , Rasmussen, J. , & Smol, J. P. (2010). *A foundation for the future: Building an environmental monitoring system for the oil sands region*. http://caid.ca/RepEnvMonOilSan2010.pdf

[ieam4485-bib-0002] Dubé, M. G. , Dunlop, J. , Davidson, C. , Beausoleil, D. L. , Hazewinkel, R. , & Wyatt, F. (2021). History, overview and governance of environmental monitoring in the oil sands region of Alberta, Canada. *Integrated Environmental Assessment and Management*, 18(2), 319–332. 10.1002/ieam.4490 PMC9290666 34241945

[ieam4485-bib-0032] Dubé, M. , Cash, K. , Wrona, F. , Enei, G. , Cronmiller, J. , Abel, R. , Andreeff, W. , Berrade, D. , Davidson, C. , Dawson, J. , Dersh, A. , Dertien, K. , Donald, G. , Evans, M. , Fayant, K. , Gladue, B. , Gosselin, J. , Ilesanmi, Y. , Ladouceur, B. , … Zhira, M. (2018). *Oil sands monitoring program letter of agreement and operational framework* (53 pp). ISBN: 978‐1‐4601‐4236‐3.

[ieam4485-bib-0033] Ebel, B. (2014). *Prospects for Aboriginal Language in Canada in Enikȍ Sepsi, Judit Nagy, Miklós Vassányi and János Kanyeres*. *Indigenous Perspectives of North America: A Collection of Studies*. Cambridge Scholars Publishing.

[ieam4485-bib-0034] Enns, E. , Littlechild, D. , & Indigenous Circle of Experts Co‐Chairs . (2018). *We Rise Together. Achieving Pathway to Canada Target 1 though the creation of Indigenous Protected and Conserved Areas in spirit and practice of reconciliation*. http://publications.gc.ca/collections/collection_2018/pc/R62‐548‐2018‐eng.pdf

[ieam4485-bib-0035] Ferguson, J. , & Weaselboy, M. (2020). Indigenous sustainable relations: considering and in language and language in land. Current Opinion in Environmental Sustainability, 43, 1–7.

[ieam4485-bib-0036] Fernández‐Llamazares, Á. , Garteizgogeascoa, M. , Basu, N. , Brondizio, E. S. , Cabeza, M. , Martínez‐Alier, J. , McElwee, P. , & Reyes‐García, V. (2020). A state‐of‐the‐art review of Indigenous peoples and environmental pollution. Integrated Environmental Assessment and Management, 16(3), 324–341. 31863549 10.1002/ieam.4239 PMC7187223

[ieam4485-bib-0037] Foster, K. R. , Davidson, C. , Tanna, R. N. , & Spink, D. (2019). Introduction to the virtual special issue monitoring ecological responses to air quality and atmospheric deposition in the Athabasca Oil Sands region the Wood Buffalo Environmental Association's Forest Health Monitoring program. *Science of the Total Environment*, 686(10), 345–359. https://www.sciencedirect.com/journal/science‐of‐the‐total‐environment/special‐issue/10LW6CG6CPT 10.1016/j.scitotenv.2019.05.353 31181521

[ieam4485-bib-0038] Garibaldi, A. , & Straker, J. (2009). *Cultural keystone species in Oil Sands mine reclamation, Fort McKay, Alberta, Canada*. Report in British Columbia Mine Reclamation Symposium, open collection. https://open.library.ubc.ca/cIRcle/collections/59367/items/1.0042557

[ieam4485-bib-0039] Golzadeh, N. , Brast , B. D. , Baker , J. M. , Auger , J. C. , & McKinney , M. A. (2021). Alkylated polycyclic aromatic hydrocarbons are the largest contributor to polycyclic aromatic concentrations in traditional foods of the Big Stone Cree Nation in Alberta, Canada. Environmental Pollution, 275, 1116625. 10.1016/j.enpol.2021.116625 33582641

[ieam4485-bib-0040] Government of Alberta . (2017). *Ambient environmental monitoring plan for oil sands development*. http://environmentalmonitoring.alberta.ca/activities/oil‐sands‐monitoring‐projects/20172018‐projects/

[ieam4485-bib-0041] Government of Alberta . (2018). *Oil sands monitoring program annual report*. https://open.alberta.ca/publications/oil‐sands‐monitoring‐program‐annual‐report

[ieam4485-bib-0042] Government of Alberta . (2019a). *Indigenous lake monitoring program*. https://open.alberta.ca/dataset/71a29cbe‐eb13‐4c03‐9021‐3a46bf1087be/resource/64a76c42‐0050‐4d28‐a8e0‐e2e5bc296396/download/indigenous‐lakes‐monitoring‐program‐fact‐sheet.pdf

[ieam4485-bib-0043] Government of Alberta . (2019b). *Oil sands monitoring program annual report*. http://environmentalmonitoring.alberta.ca/resources/document‐library‐2/

[ieam4485-bib-0045] Government of Canada . (1968–1982). *Constitution Act*. https://laws‐lois.justice.gc.ca/eng/const/page‐13.html

[ieam4485-bib-0046] Government of Canada . (2016). *Oil Sands: Indigenous Peoples*. https://www.nrcan.gc.ca/energy/publications/18736

[ieam4485-bib-0048] Government of Canada . (2019). *Video series: Wildlife science in the Oil Sands region*. https://www.canada.ca/en/environment‐climate‐change/services/oil‐sands‐monitoring/video‐wildlife‐science‐oil‐sands.html

[ieam4485-bib-0050] Gray, C. , & Rűck, D. (2019). *Reclaiming Indigenous place names*. Yellowhead Institute. https://yellowheadinstitute.org/2019/10/08/reclaiming‐indigenous‐place‐names/

[ieam4485-bib-0051] Hallett, D. , Chander, M. J. , & Lalonde, C. E. (2007). Aboriginal language knowledge and youth suicide. Cognitive Development, 3(23), 392–399. 10.1016/j.cogdev.2007.02.001

[ieam4485-bib-0052] Henshaw, A. (2006). Pausing along the journey: Learning landscapes, environmental change and typonomy among the Sikusilarmiut. Arctic Anthropology, 40(1), 52–56.

[ieam4485-bib-0053] Hoover, E. , Cook, K. , Plain, R. , Sanchez, K. , Waghiyi, V. , & Miller, P. (2012). Indigenous people of North America: Environmental exposures and reproductive justice. Environmental Health Perspectives, 120(12), 1646–1649. 10.1289/eph.1205422 PMC3548285 22899635

[ieam4485-bib-0054] Hopkins, D. , Joly, T. L. , Sykes, H. , Waniandy, A. , John, G. , Lorrie, G. , Hansen, L. , Wall, K. , Fortna, P. , & Bailey, M. (2019). “Learning together”: Braiding Indigenous and western knowledge systems to understand freshwater mussel health in the lower Athabasca region of Alberta, Canada. Journal of Ethnobiology, 39(2), 315–336. 10.2993/0278-0771-39.2.315

[ieam4485-bib-0003] Horb, E. C. , Wentworth, G. R. , Makar, P. A. , Liggo, J. , Boutzis, E. I. , Beausoleil, D. L. , Hazewinkel, R. O. , Mahaffey, A. C. , Sayanda, D. , Wyatt, F. , & Dubé, M. G. (2021). A decadal synthesis of atmospheric emissions, ambient air quality and deposition in the oil sands region. *Integrated Environmental Assessment and Management*, 18(2), 333–360. 10.1002/ieam.4539 PMC9299045 34676977

[ieam4485-bib-0055] Indigenous Guardians Toolkit . (2021). *An evolving data management system: The [MCFN] Mikisew Cree First Nations’ Approach*. https://www.indigenousguardianstoolkit.ca/story/evolving‐data‐management‐system‐mikisew‐cree‐first‐nations‐approach

[ieam4485-bib-0056] Integrated Environmental Consultants . (2018). *Volume 1. Milestone 3—Final Strategic Environmental Assessment of Wood Buffalo National Park World Heritage Site*. https://www.ceaa‐acee.gc.ca/050/documents/p65505/122894E.pdf

[ieam4485-bib-0057] Inuit Circumpolar Council . (2013). *Application of traditional knowledge in the Arctic Council*. http://www.inuitcircumpolar.com/application‐of‐indigenous‐knowledge‐in‐the‐arctic‐council.html

[ieam4485-bib-0058] Johnson, N. , Behe, C. , Danielson, F. , Krümmel, N. , Scot, P. , & Peter, L. (2016). *Community‐based monitoring and Indigenous knowledge in a changing Arctic: A review for the Sustaining Arctic Observing Networks*. https://iccalaska.org/wp‐icc/wp‐content/uploads/2016/05/Community‐Based‐Monitoring‐and‐Indigenous‐Knowledge‐in‐a‐Changing‐Arctic_web.pdf

[ieam4485-bib-0059] Joly, T. L. , & Westman, C. N. (2019). *Taking research off the shelf: Impacts, benefits and participatory processes around the Oil Sands industry in Northern Alberta*. Final Report for SSHRC Imagining Canada's Future Initiative, Knowledge Synthesis Grants: Aboriginal Peoples. University of Sasakatchewan.

[ieam4485-bib-0060] Jones, K. (2019). Whitefish Fall Fish Camp 2019. [MCFN] Mikisew Cree First Nation, [ACFN] Athabasca Chipewyan First Nation & Smith's Landing First Nation. https://storymaps.arcgis.com/stories/11bc202070de4adb9cdca340f6a7f4e5

[ieam4485-bib-0061] Kanu, A. , Dubois, C. , Hendrikis, E. , Cave, K. , Hartwig, K. , Fresque‐Baxter, J. , Trembath, K. , & Kelly, E. (2016). *Realizing the potential of community‐based monitoring in assessing the health of our waters*. Government of Northwest Territories. http://awsassets.wwf.ca/downloads/realizing_the_potential_of_community_based_monitoring_in_assessing_the_health_of_our_.pdf

[ieam4485-bib-0062] Kouril, D. , Frugal, C. , & Whillans, T. (2016). Trends and key elements in community‐based monitoring: A systematic review of the literature with emphasis on Artic and Subarctic regions. Environmental Review, 24, 151–163. 10.1139/er-2015-004

[ieam4485-bib-0063] Lawe, L. B. , Wells, J. , & Mikisew Cree First Nation . (2005). Cumulative effects assessment and EIA follow‐up: A proposed community‐based monitoring program in the Oil Sands region, northeastern Alberta. Impact Assessment and Project Proposal, 23(3), 205–209.

[ieam4485-bib-0065] Longley, H. (2015). Indigenous battles for environmental protection and economic benefits during the commercialization of the Alberta Oil Sands, 1967–1986. In A. Keeling & J. Sandlos (Eds.), *Mining and Communities in Northern Canada: History, Politics, and Memory* (pp. 208–232). https://press.ucalgary.ca/books/9781552388044

[ieam4485-bib-0066] Low, M. , & Shaw, K. (2011). First nations rights and environmental governance: Lessons from the Great Bear Rainforest. British Columbia Studies, 172, 9–33. https://ojs.library.ubc.ca/index.php/bcstudies/article/view/2247/2311

[ieam4485-bib-0068] Mikisew Cree First Nation (MCFN) . (2015a). *Community‐based monitoring program. Polycyclic aromatic hydrocarbons (PAH) levels in the Lower Athabasca River and [PAD] Peace Athabasca Delta*. http://mikisewgir.com/cbm

[ieam4485-bib-0069] Mikisew Cree First Nation (MCFN) . (2015b). *Community based monitoring program*. http://mikisewgir.com/cbm

[ieam4485-bib-0070] Mikisew Cree First Nation (MCFN) . (2016a). Written brief of the Mikisew Cree First Nation to the Standing on Environment & Sustainable Development. House of Commons Canada. November 15, 2016. www.ourcommons.ca

[ieam4485-bib-0071] Mikisew Cree First Nation (MCFN) . (2016b). Water is everything. Nipi tapitam. An Indigenous understanding of the Outstanding Universal Value of Wood Buffalo National Park. Prepared for [UNESCO] United Nations Educational, Scientific and Cultural Organization World Heritage Centre.

[ieam4485-bib-0073] Mikisew Cree First Nation (MCFN) . (2018). Letter to Joint Review Panel, Canadian Environmental Agency. *Methodology for Impacts on Aboriginal and Treaty Rights*. https://www.ceaa‐acee.gc.ca/050/documents/p65505/122764E.pdf

[ieam4485-bib-0074] McGregor, D. (2018). From ‘decolonized’ to reconciliation research in Canada: Drawing from Indigenous research paradigms. An international Journal for Critical Geographies, 17(3), 810–831.

[ieam4485-bib-0075] McIvor, O. (2013). Language and culture as protective factors for at‐risk communities. Journal of Aboriginal Health, 5(1), 6–25. 10.18357/ijih51200912327

[ieam4485-bib-0076] Nicolas Applied Management . (1996). Northern River Basins Study Project Report No.73. Factors Affecting Future Development in Key Economic Sectors in the Peace, Athabasca and Slave River Basins. Northern River Basins Study Board. Edmonton, Alberta. ISSN 1192‐3571.

[ieam4485-bib-0077] O'Flaherty, M. , & Davidson‐Hunt, I. (2008). *Scoping exercise for Indigenous ecological classification of wetlands in the Athabasca oil sands region*. Cumulative Environmental Management Association. Fort McMurray, Alberta.

[ieam4485-bib-0006] Oil Sands Monitoring Environmental DataViewer (KISTERS) . (2021). https://aws.kisters.net/OSM/applications/public.html?publicuser=Guest#waterdata/stationoverview

[ieam4485-bib-0078] Parks Canada . (2019). Wood Buffalo National Park World Heritage Site Action Plan: Wood Buffalo National Park. http://pc.gc.ca

[ieam4485-bib-0079] Parlee, B. , Geertseman, K. , & Willier, A. (2012). Social‐ecological thresholds in a changing boreal landscape: Insights from Cree knowledge of the Lesser Slave Lake region of Alberta, Canada. Ecology & Society, 17(2), 20.

[ieam4485-bib-0080] Peace‐Athabasca‐Delta Environmental Monitoring Program . (2020). http://www.pademp.com/

[ieam4485-bib-0081] Porten, S. V. D. , Loë, R. D. , Plummer, R. , Porten, S. V. D. , Loë, R. D. , & Plummer, R. (2016). Research article: Collaborative environmental governance and Indigenous peoples: Recommendations for practice. Environmental Practice, 17(2), 134–144. 10.1017/S146604661500006X

[ieam4485-bib-0082] Reed, G. , Brunet, N. D. , Longboat, S. , & Natcher, D. (2020). Indigenous guardians as an emerging approach to Indigenous environmental governance. Conservation Biology, 35(1), 179–189. 10.1111/cobi.13532 32378218 PMC7984387

[ieam4485-bib-0004] Roberts, D. R. , Arciszewski, T. J. , Beausoleil, D. L. , Hazewinkel, R. O. , Mahaffey, A. C. , Sayanda, D. , Wyatt, F. , Bayne, E. , Dennett, J. , Fisher, J. T. , & Dubé, M. G. (2021b). A synthetic review of terrestrial biological research in the oil sands region: Ten years of published literature. *Integrated Environmental Assessment and Management*, 18(2), 388–406. 10.1002/ieam.4519 PMC9292629 34510725

[ieam4485-bib-0005] Roberts, D. R. , Hazewinkel, R. O. , Arciszewski, T. J. , Beausoleil, D. L. , Davidson, C. J. , Horb, E. C. , Sayanda, D. , Wentworth, G. R. , Wyatt, F. , & Dubé, M. G. (2021a). An integrated knowledge synthesis of regional ambient monitoring in Canada's oil sands. *Integrated Environmental Monitoring and Assessment*, 18(2), 428–441. 10.1002/ieam.4505 PMC9291055 34331737

[ieam4485-bib-0083] Sousa, A. , García‐Murillo, P. , Sahin, S. , Marlales, J. , & García‐Barrón, L. (2010). Wetland place names as indicators of manifestations of recent climate change in SW Spain (Doñana Natural Park). Climatic Change, 100, 525–557. 10.1007/s10584-009-9794-9

[ieam4485-bib-0084] Spak, S. (2005). The position of Indigenous knowledge in Canadian co‐management organizations. Athropologica, 47(2), 233.

[ieam4485-bib-0085] Straka, J. R. , & Gray, Q. Z. (2018). “We used to say rats fell from the sky after a flood”: Temporary recovery of muskrat following ice‐jams in the Peace‐Athabasca Delta. Arctic, 2(71), 218–228.

[ieam4485-bib-0086] Suter, G. W. (2007). Ecological risk assessments (2nd ed.). CRC Press.

[ieam4485-bib-0087] Thompson, K.‐L. , Lantz, T. , & Ban, N. C. (2020). A review of Indigenous knowledge and participation in environmental monitoring. Ecology and Society, 25(2), 10. 10.5751/ES-11503-250210

[ieam4485-bib-0088] Timoney, K. P. , & Lee, P. (2009). Does the Alberta tar sands industry pollute? The scientific evidence. The Open Conservation Biology Journal, 3(65), 65–81. 10.2174/18748392000903010065

[ieam4485-bib-0089] Tracking Change . (2016). Calgary (AB): University of Alberta. http://www.trackingchange.ca

[ieam4485-bib-0090] Tran, T. C. , Ban, N. C. , & Bhattacharyya, J. (2020). A review of successes, challenges, and lessons from Indigenous protected and conserved areas. Biological Conservation, 241. 10.1016/j.biocon.2019.108271

[ieam4485-bib-0091] Truth and Reconciliation Commission of Canada . (2015). Truth and reconciliation: Commission of Canada: Calls to action. http://trc.ca/assets/pdf/Calls_to_Action_English2.pdf

[ieam4485-bib-0092] United Nations Educational, Scientific and Cultural Organization . (2017). World Heritage Centre. International Union for Conservation. Mission Report.

[ieam4485-bib-0094] Wood Buffalo Environmental Association (WBEA) . (2014). *Community odour monitoring project*. Annual Report 2013–2014. No: 1410_20898_01_03. https://wbea.org/resources/reports‐publications/

[ieam4485-bib-0095] Wood Buffalo Environmental Association (WBEA) . (2015a). *Community odour monitoring project*. Annual Report 2014–2015. No: 1410_20898_02_01. https://wbea.org/resources/reports‐publications/

[ieam4485-bib-0096] Wood Buffalo Environmental Association (WBEA) . (2015b). WBEA Traditional Ecological Knowledge, fact sheet. https://wbea.org/resources/reports‐publications/program‐fact‐sheets/

[ieam4485-bib-0097] Wood Buffalo Environmental Association (WBEA) . (2016). Community odour monitoring project, Aztac. Annual Report 2015‐2016. https://wbea.org/resources/reports‐publications/program‐fact‐sheets/

[ieam4485-bib-0098] Wood Buffalo Environmental Association (WBEA) . (2019a). Traditional Knowledge. https://wbea.org/traditional‐knowledge/

[ieam4485-bib-0099] Wood Buffalo Environmental Association (WBEA) . (2019b). Community odour monitoring project. Annual Report 2018. https://comp.wbea.org/2018‐annual‐report/

[ieam4485-bib-0100] Wood Buffalo Environmental Association (WBEA) . (2020). Environmental Monitoring Services 2020–2021 Project Plan Q3 Progress Report October–December 2020. https://wbea.org/wp‐content/uploads/2021/03/WBEA‐Progress‐Report‐Q3‐Oct‐Dec‐2020.pdf

[ieam4485-bib-0101] Wood Buffalo Environmental Association (WBEA) . (2021). WBEA Terrestrial Monitoring. http://67.210.212.42/deposition/wbea‐terrestrial‐monitoring

[ieam4485-bib-0102] Westman, C. N. , & Joly, T. (2019). Oil sands extraction in Alberta, Canada: A review of impacts and processes concerning Indigenous peoples. Human Ecology, 47(1), 1330–1337. 10.1007/s10745-019-0059-6

[ieam4485-bib-0103] Wilder, B. T. , O'Meara, C. , Monti, L. , & Nabhan, G. P. (2016). The importance of Indigenous knowledge in curbing the loss of language and biodiversity. BioScience, 66(6), 449–509. 10.1093/biosci/biw026

[ieam4485-bib-0104] Wong, C. , Callegooyen, K. , Ignacm, L. , Johnson, M.‐J. (G.) , & Swanson, H. (2020). Towards reconciliation: 10 calls to action to natural scientists working in Canada. FACETS, 5, 769–783. 10.1139/facets-2020-0005

[ieam4485-bib-0105] Zurawell, R. , Adams, R. , & Emmerton, C. (2019). Wabasca Lake monitoring project results report. Government of Alberta, Ministry of Environment and Parks. https://open.alberta.ca/publications/north‐wabasca‐lake‐monitoring‐project

